# Comprehensive and advanced T cell cluster analysis for discriminating seropositive and seronegative rheumatoid arthritis

**DOI:** 10.3389/fimmu.2025.1491041

**Published:** 2025-07-24

**Authors:** Shinji Maeda, Hiroya Hashimoto, Tomoyo Maeda, Shin-ya Tamechika, Taio Naniwa, Akio Niimi

**Affiliations:** ^1^ Department of Respiratory Medicine, Allergy and Clinical Immunology, Nagoya City University Graduate School of Medical Sciences, Nagoya, Japan; ^2^ Laboratory of Biostatistics, Clinical Research Center, NHO Nagoya Medical Center, Nagoya, Japan

**Keywords:** rheumatoid arthritis, anticyclic citrullinated peptide antibodies, mass cytometry, T cell biomarker, FlowSOM, peripheral helper T cell

## Abstract

**Objective:**

Rheumatoid arthritis (RA) is classified into seropositive (SP-RA) and seronegative (SN-RA) types, reflecting distinct immunological profiles. This study aimed to identify the T cell phenotypes associated with each type, thereby enhancing our understanding of their unique pathophysiological mechanisms.

**Methods:**

We analyzed peripheral blood T cells from 50 participants, including 16 patients with untreated SP-RA, 17 patients with SN-RA, and 17 healthy controls, utilizing 25 T cell markers. For initial analysis, a dataset was established through manual T cell subset gating analysis. For advanced analysis, two distinct datasets derived from a self-organizing map algorithm, FlowSOM, were used: one encompassing all CD3+ T cells and another focusing on activated T cell subsets. Subsequently, these datasets were rigorously analyzed using adaptive least absolute shrinkage and selection operator in conjunction with leave-one-out cross-validation. This approach enhanced analysis robustness, identifying T cell clusters consistently discriminative between SP-RA and SN-RA.

**Results:**

Our analysis revealed significant differences in T cell subsets between RA patients and healthy controls, including elevated levels of activated T cells (CD3+, CD4+, CD8+) and helper subsets (Th1, Th17, Th17.1, and Tph cells). The Tph/Treg ratio was markedly higher in SP-RA, underscoring an effector-dominant immune imbalance. FlowSOM-based clustering identified 44 unique T cell clusters, six of which were selected as discriminative T cell clusters (D-TCLs) for distinguishing SP-RA from SN-RA. TCL21, an activated Th1-type Tph-like cell, was strongly associated with SP-RA’s aggressive profile, while TCL02, a central memory CD4+ T cell subset, displayed ICOS+, CTLA-4low+, PD-1low+, and CXCR3+, providing insights into immune memory mechanisms. Additionally, TCL31 and TCL35, both CD4−CD8− T cells, exhibited unique phenotypes: CD161+ for TCL31 and HLA-DR+CD38+TIM-3+ for TCL35, suggesting distinct pro-inflammatory roles. Support vector machine analysis (bootstrap n = 1000) validated the D-TCLs’ discriminative power, achieving an accuracy of 86.2%, sensitivity of 85.7%, and specificity of 80.9%.

**Conclusions:**

This study advances our understanding of immunological distinctions between SP-RA and SN-RA, identifying key T cell phenotypes as potential targets for SP-RA disease progression. These findings provide a basis for studies on targeted therapeutic strategies tailored to modulate the markers and improve treatment for SP-RA.

## 1 Introduction

Rheumatoid arthritis (RA) is a chronic autoimmune disease characterized by persistent synovitis and joint destruction (1, 2). Key diagnostic markers, anticyclic citrullinated peptide antibody (ACPA) and rheumatoid factor (RF), are pivotal in the diagnosis and prognosis of RA. ACPA targets citrullinated proteins and peptides, which are significant markers in RA (3, 4), aiding in classifying RA into seropositive (ACPA+ and/or RF+) and seronegative (ACPA− and RF−) types (5). ACPA is associated with enhanced autoimmune responses, increased proinflammatory activity, and a higher likelihood of osteoclastogenesis (5–7). Conversely, RF contributes significantly to the disease process by promoting immune complex formation and complement activation, thereby intensifying the inflammatory response (5). Therefore, both ACPA and RF are crucial indicators of joint prognosis, treatment outcomes, and risk of extra-articular complications (5). Understanding the dynamics of T cells, particularly how they associate with ACPA and RF positivity, is critical for developing novel targeted therapies for RA. This understanding helps identify patients who might benefit from specific immunomodulatory treatments.

CD4+ T helper (Th) cells play a critical role in RA pathogenesis. They exacerbate RA by promoting the production of inflammatory cytokines, leading to chronic inflammation and bone destruction (8–10). Th17 cells, a subtype of CD4+ Th cells, are notable for producing cytokines, such as tumor necrosis factor-alpha and interleukin (IL)-17, which are instrumental in driving the pathogenesis of arthritis and bone degradation (11). Th17.1 cells are resistant to regulation by regulatory T cells (Tregs) and therapies involving cytotoxic T-lymphocyte-associated antigen-4 (CTLA-4) immunoglobulin (12–14). exFoxp3 Th17 cells act as potent inducers of osteoclastogenesis under inflammatory conditions, contributing significantly to joint damage (15). IL-21-producing peripheral Th (Tph) cells are crucial in recognizing citrullinated peptides in seropositive RA (SP-RA). They support B cells in producing autoantibodies and influence disease progression, with higher levels observed in patients with SP-RA than patients with SN-RA (16, 17).

Recent research underscores the significant role of CD8 T cells in the pathogenesis of RA. In particular, clonally expanded cytotoxic CD8^+^ T cells in ACPA-positive RA recognize citrullinated antigens and contribute to synovial tissue destruction (18). Synovial CD8^+^ tissue-resident memory T cells persist in previously inflamed joints and orchestrate site-specific arthritis flares upon antigen re-encounter (19). These findings highlight the importance of understanding CD8 T cell behavior to advance therapeutic strategies. In RA, the abundance of double-negative (CD4−CD8−) T cells, particularly gamma-delta types, increases in synovial fluid. This increase highlights their distinct phenotypic and functional attributes, which are crucial for the pathogenesis of RA (20, 21). Furthermore, γδ T cells have been shown to play a critical role in the activation and inflammatory responses within the RA synovium, particularly through their involvement in cytokine production and interactions with antigen-presenting cells, suggesting their potential to exacerbate chronic inflammatory states (22, 23).

Recent studies have uncovered significant molecular defects in energy metabolism and DNA damage repair in T cells in RA. These defects impact even naïve T cells, accelerating their early senescence and promoting inflammasome activation through the mTOR pathway. Such changes exacerbate chronic inflammation and RA pathology (9, 24–26). These findings underscore the importance of a comprehensive analysis of all T cell subsets, including naïve, inflammatory effector, and double-negative T cells, to enhance our understanding of RA pathogenesis and identify prognostic biomarkers for joint destruction.

Recent studies have identified six distinct cell-type abundance phenotypes in the RA synovium, advancing our understanding of cellular composition in RA (27). This knowledge is pivotal for improving therapeutic strategies and predicting treatment responses. Understanding the correlation between these immunophenotypes and clinical outcomes, such as joint prognosis and treatment resistance, is vital for improving RA management. Although differences in CCR6+ Th cells and Tph cells between SP-RA and SN-RA in peripheral blood have been observed (16, 28), comprehensive analyses of all CD3+ T cells remain scarce. The inclusion of specific T cell dynamics in relation to serostatus (ACPA and RF) provides a direct and straightforward approach to discerning immunological factors in SP-RA, thereby facilitating the development of therapeutic strategies. This integration of serostatus with T cell behavior helps clarify why targeted therapies may succeed or fail, making it essential for improving RA management and tailoring personalized treatments. Given the invasive nature of synovial biopsies, peripheral blood analysis presents a viable, less invasive option for repeated immunological assessments crucial for this purpose.

In this study, we applied high-dimensional mass cytometry (29) in conjunction with established computational techniques for the comprehensive analysis of CD3+ T cells in SP-RA and SN-RA. This sophisticated approach allowed us to discern subtle yet meaningful differences, which can act as biomarkers, to differentiate between RA subtypes. These findings enhance our comprehension of key immunological subtleties, driving the advancement of accurate diagnostics and targeted therapeutics. The central objective of this study was to clarify the immunophenotypic differences between seropositive and seronegative RA, and to determine whether high-dimensional T cell profiling combined with advanced computational methods can robustly discriminate between these disease subtypes.

## 2 Materials and methods

### 2.1 Patients and clinical assessment

Patients newly diagnosed with RA who visited the Rheumatology Department of Nagoya City University Hospital between January 2007 and November 2019 were included in the study. Eligible patients met the 2010 American College of Rheumatology/European League Against Rheumatism classification criteria for RA. Prior to blood sample collection, none of the patients received any treatment other than abortive nonsteroidal anti-inflammatory drugs (NSAIDs). Healthy controls (HCs) were selected based on the absence of pre-existing immunological disorders, such as autoimmune diseases, inflammatory conditions, infections, and allergies.

Clinical data extracted from the participants’ medical records included age, gender, duration of illness, use of NSAIDs, duration of morning stiffness, and number of tender and swollen joints. Both patient and physician global assessments were scored using a visual analog scale (VAS) ranging from 0 to 100 mm. Laboratory measurements comprised levels of C-reactive protein (CRP), matrix metalloproteinase-3 (MMP-3), RF, and ACPA. Disease activity was assessed using disease activity score 28-joint count CRP (DAS28-CRP) and simplified disease activity index (SDAI) at the time of blood sample collection.

### 2.2 Staining protocol and peripheral T cell subset analysis by mass cytometry

**Figure 1:**
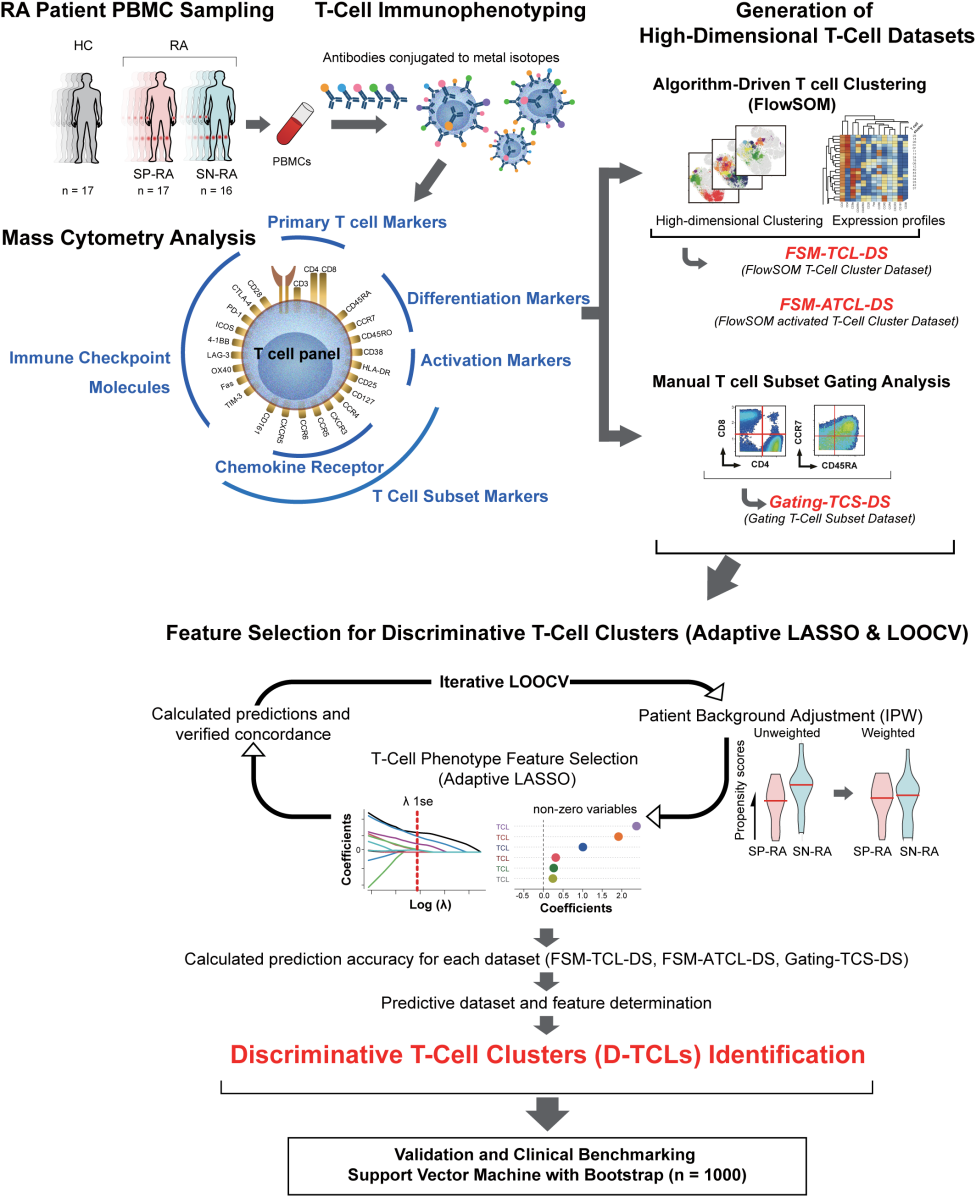
Integrative analysis workflow: from T cell profiling to discriminative cluster identification. The figure provides a comprehensive illustration of the study’s workflow, including all major datasets and abbreviations: FSM-TCL-DS (FlowSOM T cell cluster dataset: 44 clusters from all CD3+ T cells), FSM-ATCL-DS (FlowSOM activated T cell cluster dataset: 12 clusters from activated CD38+HLA-DR+ T cells), gating-TCS-DS (manually gated T cell subset dataset), D-TCLs (discriminative T cell clusters, defined as those selected by adaptive LASSO in >50% of LOOCV iterations), and ATCLs (activated T cell clusters). The relationships between datasets, feature selection, and validation steps are depicted. Starting with the collection of peripheral blood from 50 participants, including 16 patients with untreated SP-RA, 17 patients with SN-RA, and 17 healthy controls, T cells were stained for 25 markers and analyzed using mass cytometry. Initial data segmentation was achieved through manual gating of T cell subsets, followed by an advanced clustering using the FlowSOM algorithm, which created two datasets: one for all CD3+ T cells and another focusing on activated T cell subsets. These datasets facilitated the detailed examination and identification of unique T cell clusters. The number of clusters for FSM-TCL-DS and FSM-ATCL-DS was determined empirically, based on biological interpretability and hierarchical merging criteria. Subsequently, the adaptive LASSO method was applied 33 times with leave-one-out cross-validation (LOOCV), with inverse probability weighting (IPW) in each cycle for background adjustment. Clusters selected as non-zero coefficients in >50% of LOOCV cycles were defined as discriminative T cell clusters (D-TCLs). All model parameters and feature selection criteria were established *a priori*, and no *post hoc* optimization was performed, in order to minimize bias and overfitting. This analysis highlighted six D-TCLs critical for distinguishing between SP-RA and SN-RA. The identified clusters were further validated using a support vector machine (SVM) with extensive bootstrap analysis, demonstrating their significance in differentiating disease states. This integrative approach underscores the potential of detailed T cell phenotyping in uncovering nuanced immunological differences between RA subtypes and guiding targeted therapeutic strategies.

A comprehensive flowchart illustrating the methodological approach of this study is presented in **Figure 1**, summarizing the processes and analyses undertaken. Employing CyTOF analysis (26), peripheral blood (10 mL) was collected into heparin tubes from patients with RA and HCs. Peripheral blood mononuclear cells (PBMCs) were isolated using density gradient centrifugation with Leucosep tubes (Greiner Bio-One GmbH, Kremsmuenster, Austria) and Ficoll-Paque Plus (Cytiva, Tokyo, Japan) and suspended in RPMI 1640 medium enriched with L-glutamine and phenol red (FUJIFILM, Tokyo, Japan). PBMCs were cryopreserved at −80°C using Cell Banker 1 plus (Takara Bio Inc., Japan) until analysis. PBMCs were thawed in a 37°C incubator and washed with Maxpar Cell Staining Buffer (Fluidigm, South San Francisco, CA, USA). Dead cells were identified by incubation with 0.1 M cisplatin using Cell-ID Cisplatin-198Pt (Fluidigm). To prevent nonspecific binding, cells were blocked with Human TruStain FcX (BioLegend, San Diego, CA, USA). A total of 1 million cells per sample were barcoded using CELL-ID 20-plex PD Barcoding Kit (Fluidigm), following the manufacturer’s protocol. The barcoded samples were pooled for staining. Two technical control samples were incorporated into all pools to facilitate data normalization and ensure measurement consistency across analysis dates.

To profile the immunological landscape of T cells, 25 distinct cocktails of metal isotope-tagged monoclonal antibodies (Fluidigm) were prepared for cell surface staining. The target antigens included CD3, CD4, CD8, CD45RO, CD45RA, CCR7, Human leukocyte antigen-DR isotype (HLA-DR), CD38, CD25, CD127, CXCR3, CCR5, CCR4, CCR6, CD161, CXCR5, programmed death-1 (PD-1), CD28, CTLA-4, lymphocyte activation gene 3, inducible T cell costimulatory (ICOS), 4-1BB, OX40, Fas, and T cell immunoglobulin and mucin domain-containing protein 3 (TIM-3). The specifics of the metal isotope-tagged monoclonal antibodies, including antibody clones and metal isotopes, are detailed in **Supplementary Table**. One million PBMCs from each sample were stained with this antibody cocktail for 1 h at 4°C. The cells were centrifuged, washed, and fixed with 1.6% formaldehyde prepared from a 16% stock solution (w/v) (Thermo Scientific) in Maxpar PBS (Fluidigm). The fixed cell specimens were securely transported through refrigerated mail to St. Luke’s SRL Advanced Clinical Research Center, Inc. (previously known as St. Luke’s Medical & Biological Laboratories Corporation, Tokyo, Japan) for analysis. Subsequently, cell samples were incubated with 125 nM iridium intercalator (Cell-ID Intercalator-Ir 125 μM, Fluidigm) in Maxpar Fix and Perm Buffer (Fluidigm) overnight at 4°C, washed with Milli-Q water, resuspended, filtered through a 35-μm nylon mesh, and prepared with EQ Four-Element Calibration Beads (Fluidigm), according to the manufacturer’s protocol. The samples were analyzed using a Helios mass cytometer and CyTOF System (Fluidigm). Cytometry data were subsequently exported to the FCS 3.0 file format.

As a foundational component of our study, we employed a manual gating strategy to analyze the distribution of T cell subsets (**Supplementary Figure 1**). This approach was based on markers previously established in our research and those widely recognized in the field (14, 26). To ensure robust and consistent analysis, adjustments were made to account for differences in data distribution between Mass Cytometry and Flow Cytometry, aligning results with established cell subset definitions (**Supplementary Methods 2.2**). Specifically, the analysis of Treg subsets was guided by insights from recent human Treg studies (27), emphasizing the precision required in the identification and selection of these cell populations.

T cell subsets were defined based on established immunophenotypic criteria described in previous studies (30). These subsets included Th1 (CD3+CD4+CD8−CD45RO+CXCR3+CCR4−CCR6−), Th2 (CD3+CD4+CD8−CD45RO+CCR4+CXCR3−CCR6−), Th17 (CD3+CD4+CD8−CD45RO+CCR6+CCR4+CXCR3−), Th17.1 (CD3+CD4+CD8−CD45RO+CCR6+CD161+CXCR3+CCR4−), Tph (CD3+CD4+CD8−CD45RO+PD-1high+CXCR5−ICOS+), Treg (CD3+CD4+CD25+CD127−), naïve Tregs (Treg fraction I, CD45RA+CD25low+Treg), effector Tregs (Treg fraction II, CD25high+CD45RA−Treg), central memory (CM) T cells (CCR7+CD45RA−), effector memory (EM) T cells (CCR7−CD45RA−), terminally differentiated effector memory T cells re-expressing CD45RA (TEMRA) T cells (CCR7−CD45RA+), and naïve T cells (CCR7+CD45RA+).

We used a detailed strategy to categorize and analyze T cell subsets and their functional states. We quantitatively assessed the distribution of T cell types within the CD3+ T cell population, including CD4 single positive (CD4-SP), CD4−CD8− double-negative, CD4+CD8+ double-positive, and CD8 single positive (CD8-SP) cells. This analysis was supported by the assessment of central and EM cells among the CD4+ and CD8+ T cell populations, in addition to the measurement of frequencies of naïve and effector T cells within these groups.

We identified specific T cell subsets, such as Th1, Th2, Th17, Th17.1, and Tph as well as Tregs within the CD4+ lineage. Established markers were used to facilitate this classification and ensure the rigor of our gating strategy. For a deeper insight into the immunoregulatory environment, we planned to calculate the ratios of Th cells to Tregs, aiming to delineate the balance between these cell types.

This methodological setup laid the groundwork for creating gating T cell subset dataset (gating-TCS-DS), which focuses on well-characterized T cell subsets and includes a comprehensive array of immunological markers and functional characteristics pertinent to each subset. This dataset intends to serve as a bridge for subsequent analyses, including machine learning-based clustering, which will elucidate the complex immunological landscape associated with RA.

### 2.3 Unsupervised FlowSOM clustering of T cell and activated T cell clusters in RA using mass cytometry

FlowSOM, a machine-learning algorithm, was applied to CyTOF data gated on CD3+ T cells from all participants (31, 32). This approach resulted in two key cluster datasets: the FlowSOM T cell cluster dataset (FSM-TCL-DS), containing 44 clusters (TCLs, TCL00–TCL43) from all CD3+ T cells, and the FlowSOM activated T cell cluster dataset (FSM-ATCL-DS), containing 12 clusters (ATCLs, ATCL00–ATCL11) from activated CD38+HLA-DR+CD3+ T cells. Additionally, canonical T cell subsets were manually gated, forming the gating-TCS-DS. These abbreviations are used consistently throughout the manuscript. High-dimensional data visualization was performed using t-SNE, and the phenotypic profiles of each cluster were summarized in heatmaps. While some clusters appear to be closely positioned or overlap in the two-dimensional t-SNE plot, this visualization does not necessarily reflect true separation in the original high-dimensional marker space. Cluster distinctiveness was therefore further confirmed by examining comprehensive marker expression heatmaps. Details of the clustering procedure, preprocessing, and analytical methods—including cluster number selection—are provided in **Supplementary Methods 2.3**.

To ensure the robustness of clustering results and exclude potential batch effects, additional analyses—such as inter-run normalization using the CytoNorm algorithm, permutation-based multivariate analysis of variance based assessment of sample grouping, and Principal Component Analysis—were conducted. The details of these analyses are described in **Supplementary Methods 2.3.4**, and representative results are shown in **Supplementary Figure 2**.

### 2.4 Feature selection and discriminative T cell cluster identification using adaptive least absolute shrinkage and selection operator

This study utilized adaptive least absolute shrinkage and selection operator (adaptive LASSO) to identify T cell clusters that distinguish SP-RA from SN-RA. Analysis was conducted on three datasets (gating-TCS-DS, FSM-TCL-DS, and FSM-ATCL-DS), comprising immunophenotypic data from 33 RA patients stratified by ACPA status. Data normalization was performed using the centered log-ratio transformation to account for compositional biases.

To rigorously prevent overfitting and ensure unbiased performance estimation, we employed a leave-one-out cross-validation (LOOCV) framework: in each of 33 cycles, one patient was excluded as the test set, and the model was trained on the remaining 32. Within each LOOCV cycle, inverse probability weighting (IPW) was used to adjust for background covariates (age, sex, symptom duration, NSAID use). Adaptive LASSO feature selection was then performed, and clusters selected as non-zero coefficients in >50% of LOOCV iterations were designated as discriminative T cell clusters (D-TCLs). All model parameters and selection criteria were established *a priori* and applied consistently, minimizing bias and overfitting.

The workflow of the feature selection and D-TCL identification process, including LOOCV, and adaptive LASSO application, is illustrated in **Figure 1**. This visual summary highlights the analytical pipeline and key steps in identifying robust biomarkers for distinguishing SP-RA and SN-RA. Further methodological details, including statistical modeling and validation processes, are described in **Supplementary Methods 2.4**.

### 2.5 Weighted comparative analysis of T cell cluster distributions in patients with SP-RA and SN-RA

Differences in T cell cluster distributions between SP-RA and SN-RA groups were analyzed using weighted Mann–Whitney U tests, incorporating propensity score modeling and inverse probability weighting (IPW) for confounding adjustments. Weighted TCL distributions were visualized through scatter plots to provide an intuitive understanding of the comparative analysis. The detailed methodology, including IPW weight calculation, median value computation, and data visualization, is described in **Supplementary Methods 2.5**.

### 2.6 Classification performance evaluation

#### 2.6.1 Bootstrap-supported SVM validation of D-TCLs and clinical benchmarks

To evaluate the discriminative power of the identified D-TCLs in distinguishing SP-RA from SN-RA, we employed a support vector machine (SVM) model with bootstrap resampling (n = 1000 iterations).

For each bootstrap sample, the dataset was randomly split into training and test sets (typically 70% train, 30% test). The SVM hyperparameters (cost and gamma) were optimized by grid search using the training set, and model performance was assessed on the corresponding test set.

Performance metrics—including accuracy, sensitivity, specificity, positive predictive value (PPV), negative predictive value (NPV), F1 score, and area under the receiver operating characteristic curve (AUC-ROC)—were calculated for each bootstrap iteration.

Distributions of performance metrics were summarized with violin and box plots, and mean values, confidence intervals, and interquartile ranges were reported. All validation was performed internally using bootstrap-supported train/test split.

Further methodological details are provided in **Supplementary Methods 2.6**, and an overview of the analytic workflow is shown in **Figure 1**.

#### 2.6.2 Permutation test for SVM predictive performance

To further evaluate whether the observed SVM classification performance could be attributed to chance or model flexibility, we performed a permutation test. The observed mean area under the ROC curve (AUC) was calculated using 1000 bootstrap resamplings of the dataset, with SVM hyperparameters (cost and gamma) fixed to the optimal values determined on the original labels. For the permutation test, the ACPA status labels were randomly shuffled 1000 times, and for each permutation, the mean AUC was recalculated using the same SVM model and parameter settings. The distribution of permuted mean AUCs was then compared to the observed mean AUC, and a permutation p-value was determined as the proportion of permutations with mean AUCs greater than or equal to the observed value. Full implementation details are provided in **Supplementary Methods 2.6**.

#### 2.6.3 Alternative classifier validation

To further examine the robustness of the predictive signature, we performed bootstrap validation (n = 1000) using three alternative classification algorithms—Elastic Net, Random Forest, and XGBoost—in addition to the SVM. For each bootstrap sample, the optimal regularization parameter (lambda) for the Elastic Net model was determined by internal cross-validation within the training set. For Random Forest, the number of variables randomly sampled at each split (mtry) was selected by 3-fold cross-validation. For XGBoost, fixed parameters (max_depth = 3, eta = 0.1, nrounds = 50) were used based on preliminary tuning. Model performance (AUC) was evaluated on the corresponding test set. Full implementation details are provided in **Supplementary Methods 2.6**.

### 2.7 Statistical analysis

#### 2.7.1 Patient background and descriptive statistics

For evaluating differences in patient background characteristics, we employed the Mann–Whitney *U* test for continuous variables and Fisher’s exact test for categorical variables. For comparing three groups involving continuous variables, the Kruskal–Wallis test was used. All tests were assessed for statistical significance at a *p*-value of <0.05.

#### 2.7.2 T cell cluster and T cell subset analysis

Percentage of T cell cluster populations and T cell subsets, including Th cells, CD4+ T cells, and CD8+ T cells, were quantified using FlowJo software and FlowSOM v3.0.18 (29). Statistical comparisons between groups for T cell subsets were conducted using the Mann–Whitney U test, with significance set at a p-value of <0.05. To account for multiple comparisons, FDR correction (Benjamini–Hochberg method) was performed within each biologically defined group (core T cell subsets; activated T cell subsets; and Th/Treg ratio group, which includes Th and Treg frequencies as well as their calculated ratios). Both p-values and q-values are reported throughout.

#### 2.7.3 Quantitative analysis for Treg cell marker expression

Expression levels of co-stimulatory and inhibitory molecules (CD28, CTLA-4, PD-1, Fas, ICOS, LAG-3, TIM-3, OX40, HLA-DR, and 4-1BB) were quantified on total Treg cells and their functional subfractions—naïve Tregs (Fraction I) and effector Tregs (Fraction II)—using FlowJo software. For each sample, the median expression level of each marker was calculated.

Group comparisons were made between RA patients (SP-RA and SN-RA) and HCs, as well as between SP-RA and SN-RA subgroups. The Mann–Whitney U test was used to assess statistical significance. Expression data are presented as median values with interquartile ranges (IQR).

This analysis aimed to characterize the differential expression of immunoregulatory molecules across Treg subsets in RA, providing insights into their potential roles in disease-specific immune regulation.

#### 2.7.4 Correlation analysis

Spearman’s rank correlation coefficient was used to assess correlations between the proportions of each T cell population (subsets and clusters, using CLR-transformed values for compositional data) and clinical background data, including age, gender, duration of illness, morning stiffness, number of tender and swollen joints, patient VAS scores, and laboratory measures (CRP, MMP-3, RF, ACPA levels, DAS28-CRP, and SDAI). Correlation matrices were visualized to enhance the interpretability of these associations, with the Benjamini–Hochberg procedure applied to control the false discovery rate in the face of multiple comparisons (33).

All statistical analyses and visualizations were conducted using R version 4.3.1. The corrplot package was used for generating correlation plots, and the stats package was used for other statistical computations.

### 2.8 Flow cytometric analysis of additional T cell subset characteristics

To complement the CyTOF-based profiling, additional analyses were performed using conventional flow cytometry to evaluate the composition and transcription factor expression of selected T cell subsets. These included quantification of γδ T cells within the CD4^-^CD8^-^ double-negative T cell population, as well as intracellular expression of T-bet, GATA3, RORγt, and Foxp3 in Th1, Th2, Th17, Th17.1, and Treg cells. Detailed staining protocols and gating strategies are described in the **Supplementary Methods**. Representative data are shown in **Supplementary Figure 3** (transcription factor analysis) and **Supplementary Figure 4** (γδTCR analysis).

### 2.9 Ethics statement

This study was approved by the Ethics Review Committee of the Graduate School of Medicine, Nagoya City University under the approval number 60-00-0472. The date of approval was July 10, 2017. The study was conducted in accordance with the Declaration of Helsinki. Written informed consent was obtained from all patients and HCs who participated in this study.

## 3 Results

### 3.1 Baseline characteristics of patients with RA and HCs

Thirty-three patients with RA (SP-RA, n = 16 and SN-RA, n = 17) and 17 HCs were included in this study. Details of patient demographics and clinical characteristics are summarized in **Table 1**. Patient background factors were compared between SP-RA and SN-RA groups and between RA and HC groups. Median ages of HC, SN-RA, and SP-RA groups were 51, 68, and 62 years, respectively. The disease activity (DAS28-CRP and SDAI) of RA tended to be slightly higher in the SN-RA group. The rate of oral NSAID use was similar between the SN-RA (35.3%) and SP-RA groups (31.2%). There were no significant differences between SN-RA and SP-RA groups regarding the duration of illness, duration of morning stiffness, serum CRP levels, and serum MMP-3 levels. Median serum titers in the SP-RA group were 265.5 U/mL for ACPA and 99.0 IU/mL for RF. Clinical features were generally comparable between SP-RA and SN-RA, with slightly higher disease activity in SN-RA.

**Table 1:** Clinical characteristics of patients with rheumatoid arthritis and healthy controls.

Patient characteristics	RA (n = 33)		*p-value*	HCs	*p-value*
	SN-RA (n = 17)	SP-RA (n = 16)		n = 17	
	Median [IQR] or (%)	Median [IQR] or (%)	*SN-RA vs. SP-RA*	Median [IQR] or (%)	*(RA vs. HCs)*
Age, years	68 [46, 73]	62 [54, 72.5]	*0.885*	51 [38, 69]	*0.085*
Sex: male/female, (%)	8/9 (47.06/52.94)	8/8 (50.0/50.0)	*1*	8/9 (47.06/52.94)	*0.943*
Symptom duration, months	3.00 [1.47, 4.00]	1.43 [0.93, 4.75]	*0.286*	–	–
DAS28-CRP	5.00 [3.94, 5.64]	4.24 [3.49, 5.50]	*0.28*	–	–
SDAI score	31.93 [22.18, 37.46]	21.40 [13.42, 30.74]	*0.113*	–	–
Swollen Joints (0–68)	11 [6, 13]	7 [3.75, 11.25]	*0.169*	–	–
Tender Joints (0–68)	12 [9,17]	6 [3, 13.25]	*0.108*	–	–
Physician’s global assessment scores	50 [39.00, 60.00]	41 [31.75, 48.50]	*0.2*	–	–
Patient global assessment scores	52[38.00, 69.00]	60 [36.25, 70.75]	*0.885*	–	–
Morning stiffness, (h)	1.00 [0.50, 3.00]	1.50 [0.50, 3.25]	*0.487*	–	–
Concomitant NSAIDs, n (%)	6 (35.3)	5 (31.2)	*1*	–	–
CRP, mg/dL	2.17 [0.15, 6.35]	1.17 [0.38, 5.06]	*0.666*	–	–
MMP-3, ng/mL	83.00 [43.00, 242.60]	123.00 [81.42, 185.25]	*0.721*	–	–
ACPA+, n (%)	0 (0)	16 (100)		–	–
ACPA titer (U/mL)	0.60 [0.60, 0.90]	265.50 [86.32, 354.00]	*0.818* × *10^−6^*	–	–
RF+, n (%)	0 (0)	16 (100)	*0.857 × 10^−9^*	–	–
RF titer (IU/mL)	5.00 [5.00, 7.00]	99.00 [49.75, 157.75]	*0.74 × 10^−6^*	–	–

The table summarizes the clinical characteristics of the study participants, categorized into seropositive rheumatoid arthritis (SP-RA), seronegative rheumatoid arthritis (SN-RA), and healthy controls (HCs). The parameters assessed included age, sex, symptom duration, disease activity score 28-joint count C-reactive protein (DAS28-CRP), simplified disease activity index (SDAI), physician’s global assessment scores, patient global assessment scores, morning stiffness duration, and usage of nonsteroidal anti-inflammatory drugs (NSAIDs) as well as levels of C-reactive protein (CRP), matrix metalloproteinase-3 (MMP-3), rheumatoid factor (RF), and anticyclic citrullinated peptide antibody (ACPA). Data are presented as median with interquartile range (IQR) for continuous variables and as proportions (%) for categorical variables. Differences in continuous variables between SP-RA vs. SN-RA and RA overall vs. HCs were analyzed using the Mann–Whitney test. Categorical data were analyzed using the chi square test to determine statistical significance, with corresponding *p*-values displayed in the rightmost columns of the table.

### 3.2 Comparison of T cell subsets in patients with SP-RA, patients with SN-RA, and HCs

**Figure 2:**
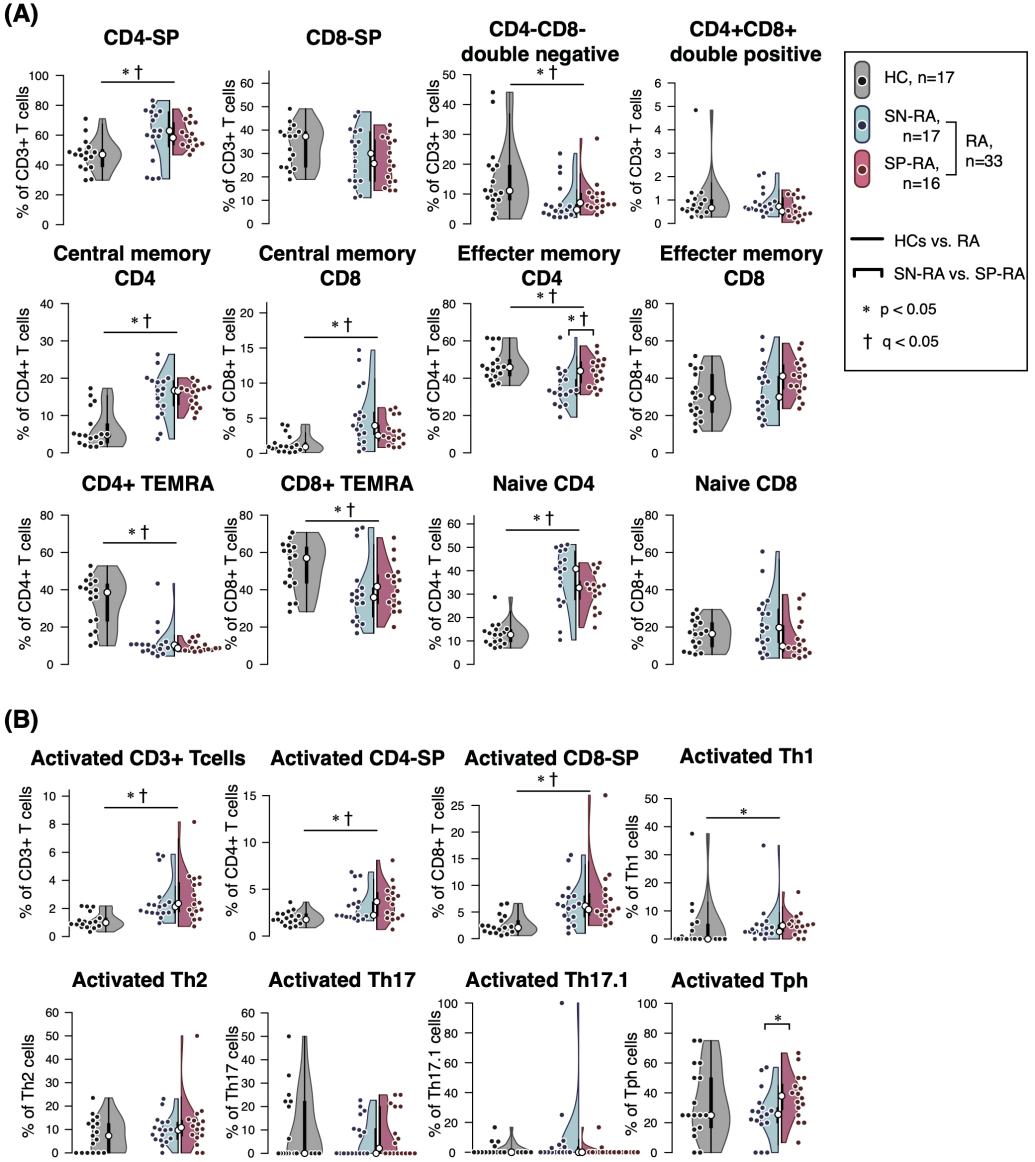
Comparative analysis of T cell subsets in seropositive and seronegative rheumatoid arthritis. The figure illustrates the proportions and ratios of various T cell subsets, excluding T helper (Th) cells and regulatory T cells (Tregs), in patients with seropositive rheumatoid arthritis (SP-RA), seronegative rheumatoid arthritis (SN-RA), and healthy controls (HCs). The analysis focuses on the relative prevalence of these subsets and their ratios, highlighting differences in immune profiles among the groups. Dot plots, violin plots, and overlaid box plots are used to display the data, showing the distribution within each group. The box plots highlight the median (indicated by a white dot) and interquartile ranges, providing a summary of the data distribution alongside the individual data points shown by the dot plots. **(A)** Core T cell subsets and **(B)** activated T cell subsets. Differences between groups were tested for statistical significance using the Mann–Whitney U test. FDR-adjusted q-values were calculated separately for the core T cell subsets (panel A) and activated T cell subsets (panel B) using the Benjamini–Hochberg method. Statistical significance is indicated by * (p < 0.05) and † (q < 0.05). Both p-values and q-values are shown for each comparison.

The peripheral blood T cell subsets were analyzed by manual gating of CyTOF data to create the gating-TCS-DS dataset (**Supplementary Figure 1**), encompassing a comprehensive set of immunophenotypic parameters detailed in **Table 2**. Significant immunological differences were observed between patients with RA and HCs. Specifically, CD4-SP, CM CD4+ T cells, CM CD8+ T cells, and naïve CD4+ T cells were significantly elevated in RA patients compared to HCs. Activated T cell subsets, including activated CD3+, activated CD4+, activated CD8+, and activated Th1 cells, were also markedly higher in RA patients (**Figure 2**, **Table 2**).

**Table 2:** Comparative analysis of T cell subsets in patients with rheumatoid arthritis and healthy controls.

		RA		HCs	*SN-RA vs. SP-RA*		*RA vs. HCs*	
T cell subset (%), Median [IQR]	ALL (n=33)	SN-RA (n=17)	SP-RA (n=16)	HCs (n=17)	*p-value*	*q-value*	*p-value*	*q-value*
Core T cell subsets								
CD4 single positive/CD3 (%)	60.00 [53.10, 70.90]	62.90 [52.20, 75.60]	58.25 [53.55, 68.28]	47.00 [38.90, 50.30]	*0.517*	*0.677*	0.00048*	*0.00144†*
CD4−CD8−-double negative/CD3 (%)	6.08 [4.10, 10.20]	4.70 [3.14, 6.44]	7.10 [5.84, 10.22]	11.10 [8.06, 19.60]	*0.084*	*0.242*	0.007*	*0.012†*
CD4+CD8+ double-positive/CD3 (%)	0.66 [0.46, 1.09]	0.72 [0.60, 0.94]	0.52 [0.32, 1.10]	0.66 [0.52, 1.01]	*0.101*	*0.242*	*0.69*	*0.75273*
CD8 single positive/CD3 (%)	26.70 [18.20, 36.70]	30.00 [18.30, 39.30]	25.70 [18.10, 35.80]	37.30 [24.20, 39.30]	*0.639*	*0.697*	*0.058*	*0.07733*
Central memory/CD4 (%)	16.70 [12.60, 18.90]	16.70 [12.60, 19.30]	16.55 [12.90, 17.38]	4.77 [2.66, 7.74]	*0.705*	*0.705*	0.0000117*	*0.00007†*
Central memory/CD8 (%)	3.22 [1.90, 5.59]	3.95 [2.67, 5.85]	2.56 [1.77, 4.02]	0.91 [0.50, 1.48]	*0.171*	*0.293*	0.000277*	*0.00111†*
Effecter memory/CD4 (%)	38.70 [33.00, 45.40]	33.40 [31.00, 39.30]	43.90 [37.60, 48.75]	45.80 [41.30, 49.90]	0.004*	*0.048†*	0.007*	*0.012†*
Effecter memory/CD8 (%)	37.50 [27.10, 45.10]	29.90 [23.10, 41.00]	41.10 [34.20, 47.10]	29.40 [21.70, 42.00]	*0.094*	*0.242*	*0.108*	*0.1296*
Naïve/CD4 (%)	34.10 [27.60, 42.70]	40.80 [27.60, 48.40]	32.70 [27.88, 35.52]	12.70 [9.71, 14.90]	*0.084*	*0.242*	0.000000167*	*0.000002†*
Naïve/CD8 (%)	13.20 [8.09, 27.10]	19.70 [11.80, 29.60]	9.74 [6.90, 20.73]	16.40 [9.40, 22.30]	*0.15*	*0.293*	*0.862*	*0.862*
TEMRA/CD4 (%)	8.78 [7.55, 10.80]	10.30 [7.55, 10.80]	8.70 [7.65, 10.97]	38.60 [23.20, 43.00]	*0.564*	*0.677*	0.00686*	*0.012†*
TEMRA/CD8 (%)	37.30 [31.50, 47.30]	35.90 [25.50, 41.00]	41.70 [32.62, 48.00]	57.00 [43.60, 62.80]	*0.368*	*0.552*	0.014*	*0.021†*
Activated T cell subsets								
Activated T cells/CD3 (%)	2.23 [1.69, 3.67]	2.12 [1.69, 2.45]	2.36 [1.75, 3.84]	0.99 [0.76, 1.31]	*0.564*	*0.752*	0.0000269*	*0.000112†*
Activated CD4+ T cells/CD4 (%)	3.06 [2.05, 4.60]	2.23 [2.05, 3.65]	3.67 [2.14, 4.66]	1.76 [1.48, 2.36]	*0.517*	*0.752*	0.00109*	*0.00291†*
Activated CD8+ T cells/CD8 (%)	6.01 [4.32, 8.01]	6.12 [4.09, 8.00]	5.47 [4.41, 8.44]	2.07 [1.31, 3.37]	*0.773*	*0.773*	0.0000281*	*0.000112†*
activated Th1/Th1 (%)	4.17 [2.25, 5.29]	2.64 [1.98, 5.00]	4.75 [2.62, 6.23]	0.00 [0.00, 5.26]	*0.22*	*0.587*	0.039*	*0.078*
activated Th2/Th2 (%)	10.00 [6.15, 13.50]	9.68 [5.79, 13.00]	10.85 [7.22, 13.93]	7.32 [0.00, 12.50]	*0.46*	*0.752*	*0.242*	*0.3872*
activated Th17/Th17 (%)	0.00 [0.00, 16.70]	0.00 [0.00, 10.50]	2.17 [0.00, 20.00]	0.00 [0.00, 22.20]	*0.727*	*0.773*	*0.85*	*0.967*
activated Th17.1/Th17.1 (%)	0.00 [0.00, 0.00]	0.00 [0.00, 3.49]	0.00 [0.00, 0.00]	0.00 [0.00, 0.00]	*0.125*	*0.5*	*0.967*	*0.967*
activated Tph/Tph (%)	30.00 [21.40, 42.90]	25.60 [20.00, 30.00]	37.95 [28.75, 45.80]	25.00 [16.70, 50.00]	0.037*	*0.296*	*0.926*	*0.967*
Th/Treg/ratio								
Th1/CD4+CD45RO+ (%)	12.40 [9.45, 16.70]	15.20 [10.30, 17.50]	10.45 [8.57, 12.92]	2.51 [1.45, 3.16]	*0.121*	*0.324*	0.000000176*	*0.000001†*
Th2/CD4+CD45RO+ (%)	8.59 [5.31, 11.90]	10.70 [8.59, 13.10]	6.26 [3.66, 7.63]	5.56 [3.71, 7.91]	0.007*	*0.021†*	*0.055*	*0.06875*
Th17/CD4+CD45RO+ (%)	1.10 [0.79, 1.71]	1.14 [0.81, 1.53]	1.04 [0.53, 1.71]	0.72 [0.46, 1.03]	*0.482*	*0.482*	0.026*	*0.06875*
Th17.1/CD4+CD45RO+ (%)	1.75 [0.83, 2.53]	1.75 [0.83, 2.10]	1.89 [0.78, 2.63]	0.64 [0.43, 1.06]	*0.589*	*0.589*	0.016*	*0.06875*
Tph/CD4+CD45RO+ (%)	2.60 [1.80, 3.78]	2.43 [1.55, 4.12]	2.77 [2.03, 3.56]	0.95 [0.69, 1.60]	*0.505*	*0.589*	0.0000679*	*0.000475†*
Treg/CD4 (%)	3.57 [2.43, 4.95]	4.84 [3.57, 5.47]	2.48 [1.93, 3.55]	5.00 [3.98, 5.88]	0.002*	*0.012†*	*0.0505*	*0.06875*
Treg fraction I/Treg (%)	12.1 [5.77, 15.9]	14.3 [11.1, 16.8]	8.085 [4.875, 14.575]	15.5 [9.68, 19.3]	*0.1390077*	*0.324*	*0.1122823*	*0.1684*
Treg fraction II/Treg (%)	17.6 [43, 22.9]	20.2 [14.9, 22.5]	20.2 [14.9, 22.5]	17.4 [10.3, 29.0]	*0.9856277*	*0.986*	*0.6448513*	*0.8383*
Th1/Treg Ratio	1.1475 [0.6193, 1.5118]	1.0392 [0.3072, 1.3760]	1.2087 [0.6941, 1.6112]	0.1224 [0.0692, 0.1910]	*0.358*	*0.537*	0.00000165*	*0.000009†*
Th2/Treg Ratio	0.6154 [0.4139, 1.0104]	0.6197 [0.4667, 1.0104]	0.5778 [0.4070, 0.9195]	0.2966 [0.1951, 0.3466]	*0.653*	*0.838*	0.0000294*	*0.000154†*
Th17/Treg Ratio	0.0983 [0.0576, 0.1361]	0.0825 [0.0576, 0.1188]	0.1060 [0.0625, 0.1651]	0.0385 [0.0272, 0.0651]	*0.407*	*0.573*	0.00173*	*0.00693†*
Th17.1/Treg Ratio	0.1413 [0.0444, 0.2288]	0.1293 [0.0262, 0.1822]	0.2000 [0.0732, 0.2753]	0.0291 [0.0166, 0.0841]	*0.117*	*0.324*	0.0161*	*0.0322†*
Tph/Treg Ratio	0.2277 [0.1616, 0.2907]	0.1775 [0.1174, 0.2712]	0.2432 [0.2211, 0.4294]	0.0583 [0.0317, 0.1102]	0.0496*	*0.124*	0.0000294*	*0.000154†*

**Figure 3:**
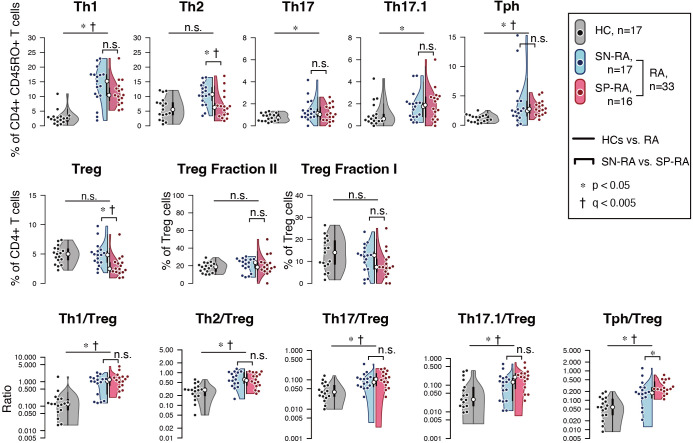
Comparative analysis of T helper and regulatory T cell profiles in seropositive and seronegative rheumatoid arthritis. **(A)** depicts the proportions of effector T helper (Th) cells and regulatory T cells (Tregs) in patients with seropositive rheumatoid arthritis (SP-RA), seronegative rheumatoid arthritis (SN-RA), and healthy controls (HCs). **(B)** examines the ratios of circulating Th1/Treg, Th2/Treg, Th17/Treg, Th17.1/Treg, and Tph cell/Treg in CD3+ T cells across three groups: SP-RA, SN-RA, and HCs. The analysis focused on the relative prevalence of these ratios, indicating differences in immune regulation across the groups. Data are presented using dot plots, violin plots, and overlaid box plots, illustrating the distribution within each group. The box plots emphasize the median (indicated by a white dot) and interquartile ranges, providing a concise summary of the data distribution while also highlighting individual data points with dot plots. Statistical significance of observed differences was assessed using the Mann–Whitney U test, and FDR-adjusted q-values (Benjamini–Hochberg method) were calculated within the Th/Treg/ratio subset group. Statistical significance is indicated by * (p < 0.05) and † (q < 0.05). Both p-values and q-values are shown for each comparison.

Data are shown as median [IQR]. The table compares various T cell subsets, including core T cell subsets, activated T cell subsets, and Th/Treg/ratio subsets, between RA subtypes and controls. The ‘Th/Treg/ratio subset group’ includes Th subset frequencies, Treg frequencies, and the calculated ratios of Th subsets to Treg. For each group, p-values were calculated using the Mann–Whitney U test, and FDR-adjusted q-values (Benjamini–Hochberg) were calculated within each group. Statistical significance is indicated by * (p < 0.05) and † (q < 0.05). See **Figures 2A, B**, and **3** for visual presentation.

Among Th subsets, proportions of Th1, Th17, Th17.1, and Tph cells were notably higher in RA patients compared to HCs (**Figure 3**, **Table 2**). Moreover, the ratios of effector Th subsets to Tregs, such as Th1/Treg, Th2/Treg, Th17/Treg, Th17.1/Treg, and Tph/Treg, were significantly elevated in RA patients, indicating a shift toward an effector-dominant immune profile. These findings highlight the distinct immunological landscape of RA compared to HCs and suggest the critical role of activated and effector T cell subsets in driving RA pathogenesis.

Further analysis within the RA cohort revealed distinct differences between SP-RA and SN-RA subgroups. While most Th subset proportions, including Th1, Th17, and Th17.1, showed no significant differences, the EM subset in CD4+ T cells tended to be higher in SP-RA than in SN-RA, reflecting a pro-inflammatory bias in the seropositive group. The activated fraction of Tph cells was significantly elevated in SP-RA compared to SN-RA (p = 0.037), consistent with its association with aggressive disease phenotypes. In contrast, the overall Treg population was significantly reduced in SP-RA compared to SN-RA (p = 0.002), suggesting impaired regulatory mechanisms in the seropositive subtype (**Figure 3**, **Table 2**). To further delineate the regulatory capacity of Tregs, their functional fractions (Fraction I and Fraction II) were examined. No significant differences in these fractions were observed between SP-RA and SN-RA groups, indicating that the observed regulatory deficit in SP-RA is driven by reduced Treg numbers rather than functional impairment.

The balance between effector Th cell and Treg was also evaluated. While the overall Th/Treg ratio was elevated in RA patients compared to HCs, only the Tph/Treg ratio showed a significant difference between SP-RA and SN-RA, being substantially higher in SP-RA (p = 0.0496). This imbalance underscores the effector-dominant immune profile characteristic of seropositive RA and its potential implications for disease progression and therapeutic targeting (**Figure 3**, **Table 2**).

To validate the immunophenotypic definitions based on surface markers used in the CyTOF analysis, we performed additional flow cytometric analyses in an independent cohort of RA patients (n = 6–8), focusing on the expression of lineage-defining transcription factors. These supplementary data, presented in **Supplementary Figure 3**, confirmed that 78% of CD4^+^CD25^+^CD127^-^ Treg cells expressed Foxp3, supporting the reliability of our gating strategy. In contrast, the expression of T-bet, GATA3, and RORγt within chemokine receptor-defined Th subsets was modest, indicating phenotypic-functional heterogeneity. These findings reinforce the validity of our Treg characterization and suggest a more variable transcriptional profile among effector T cell subsets. In addition, to address the absence of γδTCR detection in the CyTOF antibody panel, we performed supplementary flow cytometric analysis on peripheral blood samples from RA patients (n = 4). This analysis demonstrated that γδ T cells accounted for an average of 50.7% (range: 45–53%) of the CD4^-^CD8^-^ double-negative T cell population. These findings, as shown in **Supplementary Figure 4**, indicate that γδ T cells represent a substantial component of the double-negative T cell compartment in RA. These results demonstrate an effector-dominant T cell profile and reduced Treg presence in RA, especially in SP-RA.

### 3.3 Differential expression of co-stimulatory and inhibitory molecules in Treg subsets between RA and healthy controls

To clarify the phenotypic characteristics of Treg subsets in RA, we analyzed the expression of ten co-stimulatory and inhibitory surface molecules—including CD28, CTLA-4, PD-1, Fas, ICOS, LAG-3, TIM-3, OX40, HLA-DR, and 4-1BB—in total Tregs as well as their functional subfractions: naïve Tregs (Fraction I) and effector Tregs (Fraction II). The expression profiles were compared among SP-RA, SN-RA, and HCs (**Table 3**). Compared with HCs, RA patients showed significantly elevated expression of all molecules except LAG-3, across total Tregs (p < 0.05). Notably, in naïve Tregs, CD28, Fas, and ICOS were significantly upregulated, while in effector Tregs, increased expression of CD28, Fas, PD-1, and 4-1BB was observed. Between RA subtypes, CTLA-4 expression was significantly higher in SN-RA than in SP-RA within total Tregs (p = 0.035), and 4-1BB was significantly increased in naïve Tregs of SN-RA. No differences were found in effector Tregs between subtypes. These findings indicate that RA is associated with enhanced activation of Tregs, and that SN-RA may retain a more immunoregulatory Treg phenotype, particularly within the naïve compartment. This enhanced Treg activation in SN-RA may contribute to its distinct immunological profile.

**Table 3:** Expression of co-stimulatory and inhibitory molecules on treg cells in RA and healthy controls.

Treg subset	Molecule	HC	SN-RA	SP-RA	RA vs HC (p)	SN-RA vs SP-RA (p)
*Treg*	CD28	28.9 [27, 33.7]	51 [43.6, 61.9]	53.05 [43.38, 58.48]	<0.001*	0.773
	CTLA-4	1.3 [1.19, 1.54]	2.16 [1.74, 2.88]	1.5 [0.98, 2.13]	0.026*	0.035*
	Fas	12.3 [8.3, 16.8]	26.1 [15.3, 37.9]	28.3 [23.27, 39.6]	<0.001*	0.494
	HLA-DR	0.03 [0, 0.25]	0.66 [0.32, 1.66]	0.87 [0.24, 1.46]	0.004*	0.914
	ICOS	4.1 [2.12, 5.02]	5.91 [3.63, 7.76]	6.03 [4.93, 7.4]	0.001*	0.857
	LAG-3	0 [0, 0]	0 [0, 0]	0 [0, 0]	0.205	0.47
	OX40	0.15 [0, 0.44]	0.39 [0.11, 0.67]	0.49 [0.22, 1.14]	0.023*	0.407
	PD-1	0.73 [0.17, 0.9]	1.17 [0.8, 1.52]	1.48 [0.74, 1.75]	0.001*	0.601
	TIM-3	0 [0, 0]	0 [0, 0.17]	0 [0, 0.33]	0.049*	0.522
	4-1BB	0.22 [0, 0.38]	0.98 [0.53, 1.3]	0.58 [0.07, 0.75]	0.001*	0.149
*Treg Fr.I*	CD28	23 [19.5, 31.5]	37.2 [27.65, 42.3]	33.8 [26.3, 35.1]	0.01*	0.695
	CTLA-4	0.98 [0.48, 1.99]	2.62 [1.33, 3.33]	1.57 [0.38, 2.53]	0.228	0.174
	Fas	0.03 [0, 2.78]	2.6 [0.9, 5.04]	2.41 [0.06, 4.44]	0.034*	0.818
	HLA-DR	0 [0, 0.27]	0 [0, 0.03]	0 [0, 0]	0.241	0.404
	ICOS	0.33 [0, 2.32]	2.1 [1.52, 3.14]	2.81 [1.91, 5.42]	0.028*	0.269
	LAG-3	0 [0, 0.07]	0 [0, 0.2]	0.18 [0, 0.48]	0.248	0.272
	OX40	0 [0, 0]	0 [0, 0.33]	0 [0, 0.12]	0.346	0.452
	PD-1	0.26 [0, 0.87]	0.08 [0, 0.39]	0 [0, 0.78]	0.576	0.472
	TIM-3	0 [0, 0]	0 [0, 0]	0 [0, 0]	0.67	0.469
	4-1BB	0 [0, 0.61]	0.64 [0.12, 1.39]	0 [0, 0.08]	0.429	0.008*
*Treg Fr.II*	CD28	49.3 [33.9, 57.7]	77.5 [68.48, 100.62]	83.9 [65.2, 89.35]	<0.001*	0.502
	CTLA-4	1.99 [1.46, 2.69]	2.54 [1.98, 3.63]	1.89 [1.21, 3.71]	0.438	0.277
	Fas	38.3 [29.8, 48.3]	92.15 [52.72, 104.25]	95.6 [51.6, 124.5]	0.001*	0.502
	HLA-DR	2.4 [0.58, 5.81]	5.37 [3.25, 10.63]	9.35 [1.17, 31.95]	0.101	0.526
	ICOS	12.1 [7.97, 17.9]	20.8 [8.55, 24.07]	20.8 [6.67, 22.5]	0.149	0.828
	LAG-3	0 [0, 0]	0 [0, 0.09]	0 [0, 0]	0.476	0.666
	OX40	0.8 [0.46, 1.56]	1.54 [1.19, 2.08]	1.74 [0.83, 2.99]	0.051	0.565
	PD-1	0.4 [0, 1.59]	1.52 [1.11, 3]	2.54 [1.34, 4.58]	0.009*	0.323
	TIM-3	0.21 [0, 0.77]	0.65 [0, 1.18]	0.77 [0, 1.5]	0.227	0.716
	4-1BB	0.46 [0, 1.03]	0.98 [0.37, 1.84]	1.89 [1.23, 2.29]	0.006*	0.063

Expression of co-stimulatory and inhibitory molecules on Treg cells and their subfractions in patients with seropositive RA (SP-RA), seronegative RA (SN-RA), and healthy controls (HC). The table displays median values and interquartile ranges (IQR) for each molecule. For each sample, the median expression level was quantified using FlowJo software. Statistical comparisons of expression levels between different groups were performed using the Mann-Whitney test, assessing differences between RA patients (combining SN-RA and SP-RA) versus HCs, and within RA between SN-RA and SP-RA. This analysis aims to identify significant differences in the immunoregulatory profiles of Treg cells between the patient cohorts and healthy individuals. * indicates statistical significance (p < 0.05).

### 3.4 Correlation of T cell subsets with clinical background factors

**Figure 4:**
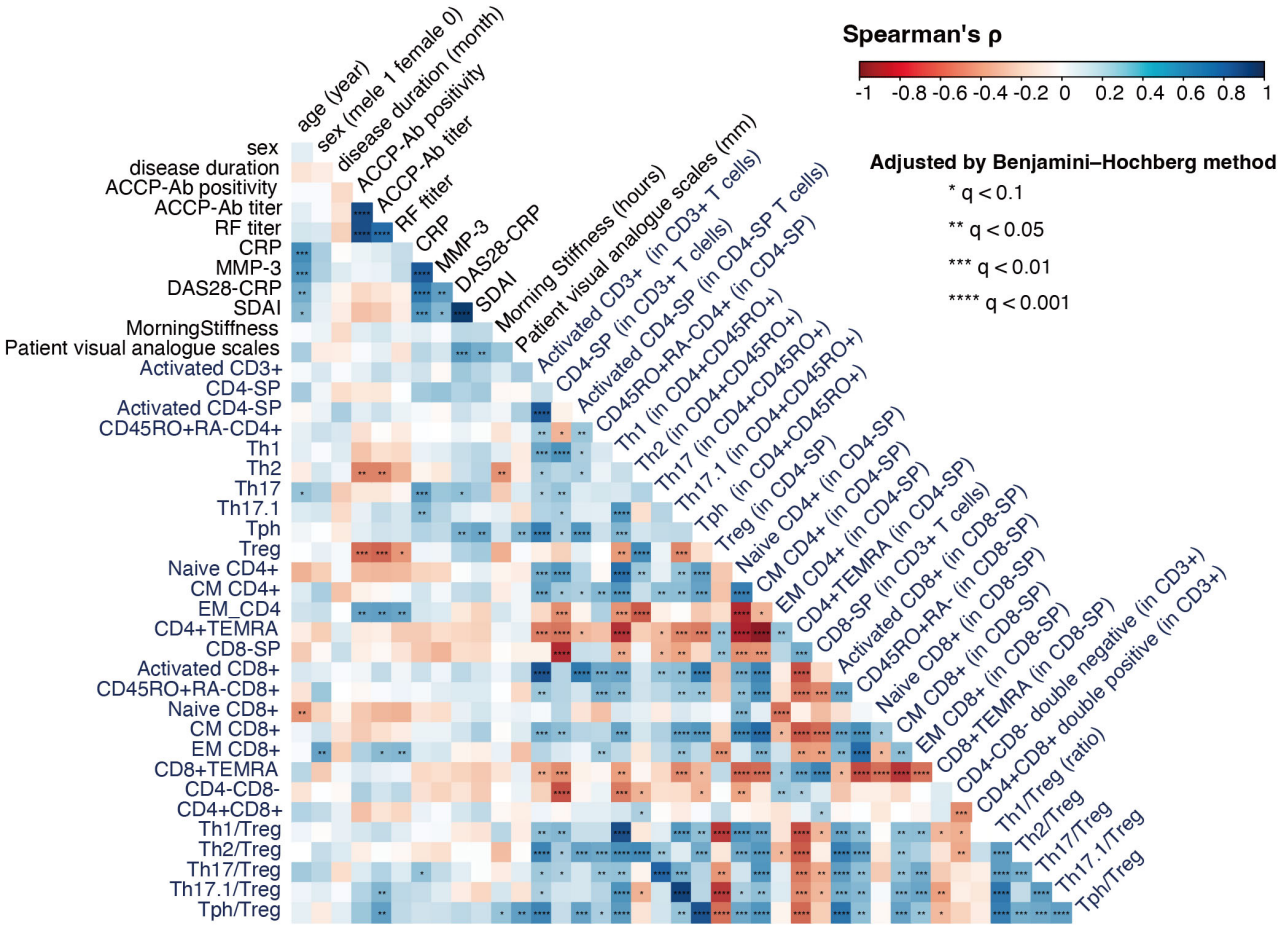
Correlation analysis of T cell subsets and clinical characteristics in rheumatoid arthritis (RA) (n = 33). The figure shows a correlation coefficient matrix (Spearman’s ρ) between T cell subset frequencies, Th/Treg ratios, and clinical background factors such as age, sex, symptom duration, and ACPA positivity. For compositional variables (e.g., T cell clusters, T cell subset frequencies), correlations were calculated using CLR-transformed values; for ratios and other non-compositional variables, raw values were used. Each cell in the matrix indicates the strength and direction of the correlation, on a scale of −1 (strong negative correlation) to +1 (strong positive correlation), represented by a color gradient from red to blue. FDR correction (Benjamini–Hochberg method) was applied for multiple testing. Significance levels are indicated within each cell using asterisks to denote Benjamini–Hochberg adjusted q-values: *q < 0.1, **q < 0.05, ***q < 0.01, and ****q < 0.001.

We next analyzed the correlations between T cell subsets and clinical background factors to further elucidate the immunological landscape in RA. This analysis, conducted using samples from RA patients (n = 33), revealed significant associations that highlight the interplay between immune cell populations and disease characteristics (**Figure 4**).

Notably, ACPA titers were negatively correlated with Th2 cells and Tregs, while positive correlations were observed with EM-CD4 and EM-CD8 cells and the Th17.1/Treg and Tph/Treg ratios. These findings underscore the effector-dominant immune profile associated with seropositive RA, characterized by a reduction in regulatory mechanisms.

Inflammatory markers, such as serum CRP levels, were positively correlated with Th17 and Th17.1 cells, as well as the Th17/Treg ratio, reinforcing the critical role of Th17-mediated inflammation in RA pathogenesis. Additionally, disease activity indices showed distinct associations with T cell subsets. DAS28-CRP was positively correlated with Th17 and Tph cells, while SDAI demonstrated a strong positive correlation with Tph cells, suggesting their potential as biomarkers for disease monitoring.

These correlations provide valuable insights into the distinct immune mechanisms driving RA subtypes and emphasize the potential clinical utility of targeting specific effector and regulatory T cell populations. Taken together, our findings further highlight the immunological divergence between SP-RA and SN-RA.

### 3.5 Dimensionality reduction plot of high-dimensional CyTOF Data for 25 markers

High-dimensional concatenated data for 25 T cell markers from the 50 participants were visualized as two-dimensional plots using t-SNE (**Supplementary Figures 5**, **6**). The expression of the 25 cell surface markers was displayed as a heatmap on the t-SNE map (**Supplementary Figure 5A**). The t-SNE maps for the HCs, SP-RA, and SN-RA groups were displayed (**Supplementary Figure 7A**). The pseudocolor plot of cell density distribution indicated differences in cell density distribution across the three groups. Additionally, on the t-SNE map, the indicated T cell subsets were presented as overlays in the indicated colors (**Supplementary Figure 7B**). The analyzed Th subset accounted for a small portion of CD3+ T cells, whereas the remaining CD4+ and CD8+ T cells could be further divided into smaller cell populations. These visualizations revealed distinct distribution patterns of T cell populations across SP-RA, SN-RA, and HC groups, supporting further phenotypic clustering.

### 3.6 T cell clustering analysis using self-organizing maps

To identify T cell clusters that distinguish SP-RA from SN-RA, we first performed unsupervised clustering using the FlowSOM algorithm, generating the FSM-TCL-DS (44 TCLs, TCL00–TCL43; see **Figure 1** for workflow). This analysis was complemented by manual gating of canonical T cell subsets (gating-TCS-DS) and FlowSOM clustering on activated T cells (FSM-ATCL-DS). Adaptive LASSO with leave-one-out cross-validation (LOOCV) and inverse probability weighting (IPW) was applied to each dataset to select discriminative T cell clusters (D-TCLs), defined as clusters consistently selected in >50% of LOOCV iterations. The relationships between datasets, cluster selection steps, and validation analyses are summarized in **Figure 1**.

The clusters derived from the FSM-TCL-DS dataset were visualized on a t-SNE map with distinct colors (**Supplementary Figure 5B**), and their marker expression profiles were summarized in a heatmap (**Supplementary Figure 5C**). We then quantified the proportion of each TCL within CD3^+^ T cells in individual samples in order to explore differences in cluster distribution between SP-RA and SN-RA.

### 3.7 FlowSOM clustering of activated T cell subsets in CD38+HLA-DR+CD3+ T cells

Similar to our approach with T cell clusters, we performed clustering using the FlowSOM algorithm on the activated T cell gate, focusing on the CD38+HLA-DR+CD3+ T cell population for further insights. In our analysis, we identified 12 distinct ATCLs within this population (**Supplementary Figure 6A**). These exhibited a wide range of immunophenotypes, as evidenced by our comprehensive heatmap analysis of 25 surface markers for each cluster (**Supplementary Figure 6B**). Notably, the ATCLs consisted of various T cell subsets: five clusters (ATCL01–03, 05, and 06) were derived from CD4+ T cells; three (ATCL09–11) from CD8+ T cells; one (ATCL00) cluster showed weak CD8 expression; and three (ATCL04, 07, and 08) were identified as originating from CD4−CD8− double-negative T cells. We further analyzed the proportion (%) of these ATCLs within CD3+ T cells for each sample, resulting in the creation of FSM-ATCL-DS. Among the 12 ATCLs, eight clusters (ATCL02, 03, 04, 05, 06, 09, 10, and 11) were found to be significantly increased in RA patients compared to HCs (**Supplementary Figure 6C**). Although no statistically significant differences were observed between SN-RA and SP-RA, ATCL02 and ATCL03 tended to be more abundant in SN-RA, while ATCL05, 06, and 09 showed a trend toward higher expression in SP-RA.

### 3.8 Identification and characterization of distinct T cell clusters in RA subtypes

Prior to identifying D-TCLs, we performed extensive analysis to ensure the robustness and reliability of our findings. We analyzed T cells from 16 patients with untreated SP-RA and 17 patients with SN-RA, utilizing mass cytometry to assess 25 T cell markers. The FlowSOM algorithm facilitated the identification of 44 T cell clusters (TCL00–43).

**Figure 5:**
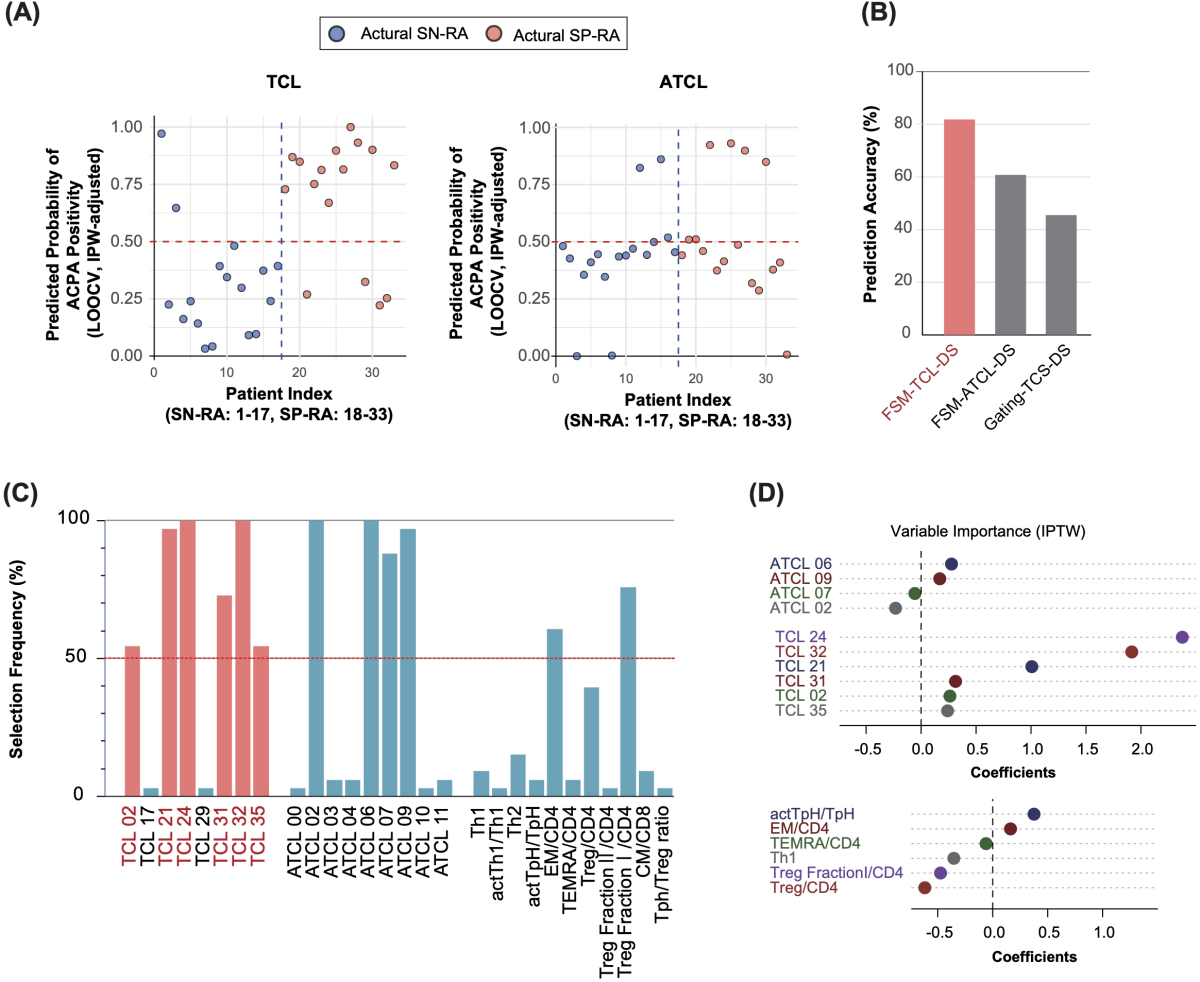
Adaptive LASSO-driven selection and visualization of discriminative T cell clusters in seropositive and seronegative rheumatoid arthritis. **(A)** Predicted probability of ACPA positivity for each patient. The y-axis shows the IPW-adjusted probability of ACPA positivity as estimated by the adaptive LASSO model using leave-one-out cross-validation (LOOCV). The x-axis represents patient IDs, indexed such that actual SN-RA cases are labeled from 1 to 17 and actual SP-RA cases from 18 to 33. Each point represents an individual patient, with predicted probabilities derived from the model trained on all other patients (see Methods for details). Predictions are adjusted using inverse probability weighting (IPW) based on patient background variables, such as sex, age, symptom duration, NSAID usage, and DAS28-CRP. A horizontal reference line at the 0.5 probability threshold clearly differentiates predictions above (ACPA-positive) from those below (ACPA-negative), providing a direct visual comparison of predicted versus actual ACPA status. **(B)** Predictive accuracy across datasets. The bar graph presents the predictive accuracy of the adaptive LASSO model the FSM-TCL-DS, FSM-ATCL-DS, and gating-TCS-DS datasets. It shows the proportion of samples correctly predicted as SP-RA or SN-RA, demonstrating the effectiveness of the model. The FSM-TCL-DS dataset achieved the highest predictive accuracy (81.8%), underscoring its utility in model validation. **(C)** Frequency of selection for T cell variables across datasets. The graph illustrates how frequently different T cell variables were selected as significant discriminators between SP-RA and SN-RA, across multiple rounds of LOOCV. Variables consistently selected in >50% of the rounds are defined as discriminative T cell clusters (D-TCLs), with clusters such as TCL02, 21, 24, 31, 32, and 35 identified as particularly influential. **(D)** Coefficients of T cell variables from adaptive LASSO analysis. The plot displays the coefficients assigned to various T cell variables through adaptive LASSO analysis performed on the entire dataset of 33 patients with rheumatoid arthritis, illustrating the relative importance of each variable in distinguishing between patient groups.

To examine the dataset, we conducted a series of analyses on three distinct datasets: gating-TCS-DS, FSM-TCL-DS, and FSM-ATCL-DS (**Figures 5A, B**). Each of these datasets underwent a specific set of analytical procedures. Importantly, for each iteration of LOOCV, we applied IPW to adjust for variations in patient backgrounds. This approach ensured that patient background adjustments were individually tailored for each LOOCV iteration. Subsequently, we used adaptive LASSO in the LOOCV process, conducted 33 times (**Figure 1**).

This rigorous approach was designed to extract features that were consistently observed in >50% of the validations, ensuring results reliability.

To determine the dataset with the highest predictive accuracy, we calculated prediction accuracy for each dataset using LOOCV. The accuracies were 81.8% for FSM-TCL-DS, 60.6% for FSM-ATCL-DS, and 45.5% for gating-TCS-DS (**Figure 5B**). Based on these outcomes, we focused on FSM-TCL-DS, which exhibited the highest accuracy. Within FSM-TCL-DS, we identified certain features that were selected as non-zero coefficients in more than 50% of LOOCV cycles (**Figure 5C**) were designated as discriminative T cell clusters (D-TCLs), reflecting robust and reproducible discriminative power and minimizing the risk of overfitting. This refined approach emphasizes the thoroughness of our analytical process, ensuring that each step is optimally aligned to enhance the validity of our findings. For additional insight into the methodological rigor of our study, we graphically demonstrated the adjustments made to propensity scores using IPW across all cases, highlighting the impact of patient background adjustments (**Supplementary Figure 8A**). Following this, our use of the adaptive LASSO model is illustrated, which visualizes the selection of significant variables from the FSM-TCL-DS, FSM-ATCL-DS, and gating-TCS-DS datasets (**Supplementary Figure 8B**). Subsequently, we displayed the T cell variables and their corresponding coefficients that were identified as non-zero variables by applying the adaptive LASSO model to the entire dataset (**Figure 5D**).

**Figure 6:**
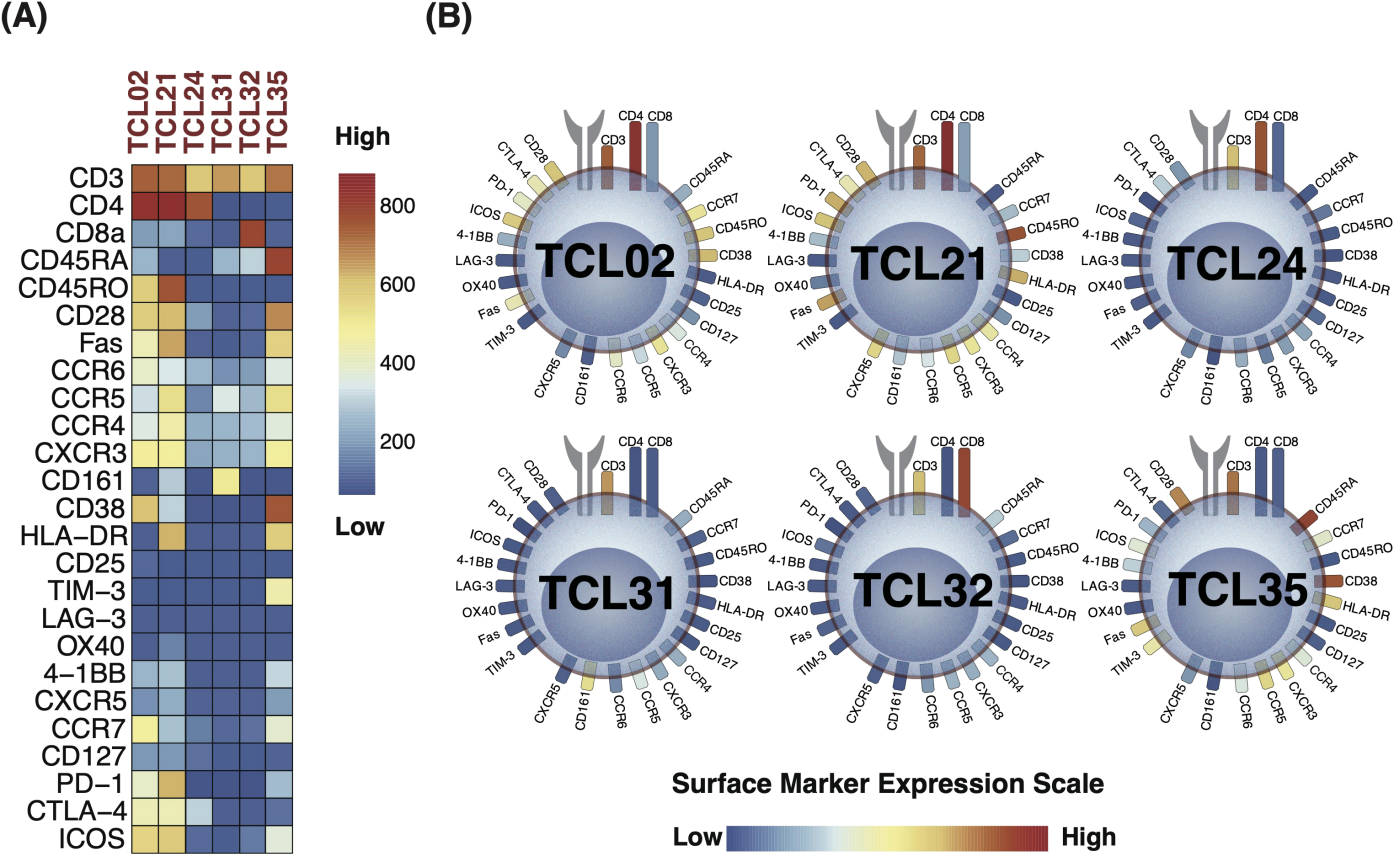
Marker expression profiles of discriminative T cell clusters (D-TCLs) identified between seropositive (SP-RA) and seronegative rheumatoid arthritis (SN-RA). **(A)** Heatmap displaying the expression levels of all measured surface markers across the six D-TCLs, using a unified global color scale (see color bar). This enables direct quantitative comparison of marker expression across clusters. The heatmap is extracted from the comprehensive 44-cluster heatmap shown in **Supplementary Figure 5C**. **(B)** Cell diagram representation of the same D-TCLs, where T cell surface markers are depicted as soft-edged rectangles, and colors correspond to their expression levels (low: navy, high: red). This diagram provides an intuitive overview of the phenotypic profiles within each cluster. Presenting both the heatmap and the cell diagram allows for both precise quantitative comparison and rapid visual assessment of the marker expression patterns of D-TCLs. The full heatmap of all 44 clusters is provided in **Supplementary Figure 5C**.

The identified D-TCLs included TCL02, TCL21, TCL24, TCL31, TCL32, and TCL35. To facilitate interpretation, marker expression profiles for these clusters are visualized using both a heatmap with a unified global color scale (**Figure 6A**) and the original cell diagram representation (**Figure 6B**). The heatmap enables direct quantitative comparison of marker expression across the D-TCLs, while the cell diagram provides an intuitive overview of the phenotypic profiles. The full heatmap of all 44 clusters is provided in **Supplementary Figure 5C**.

Each cluster displayed unique characteristics and patterns, distinct from the remaining data. Among these, TCL21 stood out as a representative of the activated Th1-type Tph cell, characterized by a specific marker expression profile. TCL21 belongs to EM CD4+ T cells and was characterized as CXCR3+CCR6−CCR5low+PD-1 high+ICOS+CD28+Fas+HLA-DR+. This cell population is considered similar to the Th1-type activated Tph cell. TCL21 displayed a positive correlation with DAS28-CRP and patient VAS. TCL02 was another significant finding, indicative of CM CD4+ T cells. The profile of this cluster was CD45RO+CD28+CD38+HL-DR−CCR7+ICOS+CTLA-4low+PD-1low+CXCR3+, suggesting a state of partial activation and memory potential. We identified two CD4−CD8− double-negative T cell clusters, TCL31 and TCL35. TCL31 was distinguished by the expression of CD161+, typically associated with innate-like T cell functions, whereas TCL35 was characterized by HLA-DR+CD38+TIM-3+, markers often associated to an activated, potentially regulatory phenotype.

Two TCLs with low T cell phenotype expression, TCL24 and TCL32, were pivotal in the stratification of patients with SP-RA and SN-RA. TCL24 is a CD4-SP cluster that is negative for CD45RA, CD45RO, and CCR7. There was little expression of other lineage markers, no expression of activation markers, and low expression of the CTLA-4 costimulatory/coinhibitory marker. TCL32 is a CD8-SP cluster that is negative for CD45RA, CD45RO, and CCR7. No other characteristic markers were identified. Although these clusters had less pronounced marker expression, they contributed significantly to the overall T cell landscape and its association with RA subtypes.

These D-TCLs may represent distinct peripheral immunophenotypes that aid in differentiating between seropositive and seronegative RA.

### 3.9 Correlation analysis of T cell clusters with clinical parameters in patients with RA: identifying signatures of disease activity and serostatus

To complement the primary findings of T cell clusters favoring SP-RA (all with coefficient values of >0), we expanded our investigation to explore associations with seronegative RA. We performed a correlation analysis between T cell clusters and a range of clinical parameters—age, gender, disease duration, disease activity scores (DAS28-CRP and SDAI), patient-reported VAS, and serum markers (CRP, MMP-3, ACPA, and RF)—in a cohort of 33 patients with RA (**Supplementary Figure 9**). This analysis aimed to delineate T cell profiles unique to seronegative RA, thus broadening our understanding of RA serostatus diversity.

Correlation analysis revealed that in addition to primary clusters (TCL24 and TCL32), TCL10 and TCL29 were significantly correlated with ACPA, predominantly in patients with SN-RA. TCL10, a CD4-SP ATCL, expressed high levels of activation markers, including HLA-DR+ and CD38+. This cluster was further characterized by a comprehensive marker profile: CD45RO+, CD45RA+, CCR7+, CXCR3high+, CXCR5+, CCR4+, CD28+, ICOS+, CTLA-4+, Fas+, and PD-1+, with lower expression of CD25 and CD127, indicating a highly activated state. Conversely, TCL29, a naïve CD4-SP T cell cluster, was marked primarily by CD45RA+ and CCR7+, denoting its naïve status. This cluster exhibited activation markers, such as CD38 and CD28, and chemokine receptors, including CXCR3 and CCR4. CCR6 expression was detected at low levels, whereas CD161 was highly expressed, suggesting a distinctive profile of the naïve T cell population in SN-RA.

Shifting our analysis to broader RA disease activity, DAS28-CRP was positively correlated with TCL21. By contrast, TCL14, an EM CD4+ T cell cluster characterized by CD45RO+, PD-1+, CCR5+, and CXCR3+, was negatively correlated with SDAI. TCL18, a CD45RO−CD45RA−CCR7− CD4-SP cluster characterized by CTLA-4low+, ICOSlow+, Faslow+, CD28+, CD25low+, CD127−, and CCR4+, mirrored the attributes of nonregulatory, proinflammatory cytokine-secreting cells (human Treg fraction III (34) and positively correlated with SDAI, CRP, and MMP-3 levels, suggesting associations with markers of RA disease activity.

**Figure 7:**
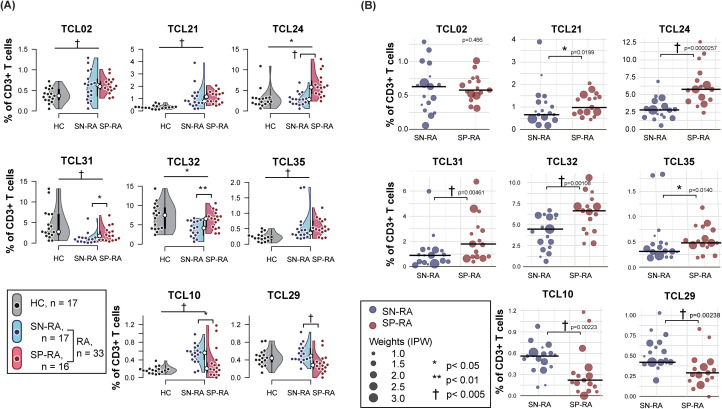
Comparative analysis of key T cell clusters in rheumatoid arthritis subtypes and healthy controls. **(A)** Non-weighted distribution of key T cell clusters (TCLs). The panel presents a combination of violin and dot plots illustrating the percentage distribution of selected T cell clusters within CD3+ T cells across three groups: seropositive RA (SP-RA), seronegative RA (SN-RA), and healthy controls (HCs). The plot features eight key T cell clusters, including six discriminative T cell clusters (D-TCLs): TCLs 02, 21, 24, 31, 32, and 35, in addition to TCL 10 and TCL 29, which have been identified through correlation analysis as having significant negative associations with ACPA positivity. The plots provide a visual comparison of the frequencies of these TCLs, highlighting variations between HCs and the combined RA groups (SN-RA and SP-RA) as well as directly between the SP-RA and SN-RA groups. Statistical significance of the differences was assessed using the Mann–Whitney *U* test, with symbols indicating levels of significance: ^*^ *p* < 0.05, ^**^ *p* < 0.01, and ^†^ *p* < 0.005. **(B)** Weighted scatter plot of key T cell clusters in patients with RA. The panel features a weighted scatter plot showing the distribution of the same eight key T cell clusters (TCLs), specifically among patients with RA, divided into the SP-RA and SN-RA groups. The sizes of the points are proportional to inverse probability weighting (IPW), which adjusts for patient background factors, such as age, sex, symptom duration, DAS28-CRP, and NSAID usage. Weighted median values for each TCL are depicted with horizontal bars. The significance of the differences, assessed using the weighted Mann–Whitney test adjusted for IPW, is marked by ^*^ *p* < 0.05, ^**^ *p* < 0.01, and ^†^ *p* < 0.005, providing detailed visualization of intercluster variability.].

In this secondary analysis, our investigation expanded to include specific T cell clusters, such as TCL10 and TCL29, newly identified through their negative correlations with ACPA. TCL21, previously identified as one of the D-TCLs, was associated with RA disease activity, suggesting its relevance in the pathogenesis of SP-RA. To illustrate the distributional differences of these T cell clusters across HCs, SP-RA, and SN-RA, a non-weighted graph was constructed (**Figure 7A**). We used IPW to evaluate the distinction in T cell distribution between SP-RA and SN-RA while adjusting for patient background. The balloon sizes in the subsequent graph represent IPW weights, visually indicating the weighted statistical significance of TCL distribution across patient groups (**Figure 7B**).

Significant variances were observed across all six D-TCLs between HCs and RA groups. The proportions of TCL31 and TCL32 were lower in patients with RA than in HCs, whereas the remaining D-TCLs were more prevalent in RA. In IPW analysis comparing SP-RA with SN-RA, except for TCL02, all other D-TCLs demonstrated significant predominance in the SP-RA group. TCL21, TCL24, and TCL35 significantly increased from HCs to RA and from SN-RA to SP-RA, underscoring their potential pathogenic significance in SP-RA. Although the differentiation between SP-RA and SN-RA was unclear, TCL02 was consistently present across all RA conditions.

Unlike the D-TCLs primarily featured in SP-RA with positive coefficients, TCL10 and TCL29 were more commonly observed in SN-RA. TCL10 exhibited higher proportions in RA than in HCs, whereas the distinction for TCL29 was not apparent. Nevertheless, both clusters were significantly increased in SN-RA compared with SP-RA. These findings further underscore the immunophenotypic diversity between SP-RA and SN-RA and its clinical relevance.

### 3.10 Internal performance assessment of D-TCLs using bootstrap-supported SVM

The discriminative performance of the identified D-TCLs for classifying SP-RA versus SN-RA was assessed using support vector machine (SVM) modeling with bootstrap-supported internal validation (n = 1000 iterations).

For each bootstrap sample, the data were randomly divided into training and test sets, the SVM was optimized via grid search, and performance was evaluated on the test set.

**Figure 8:**
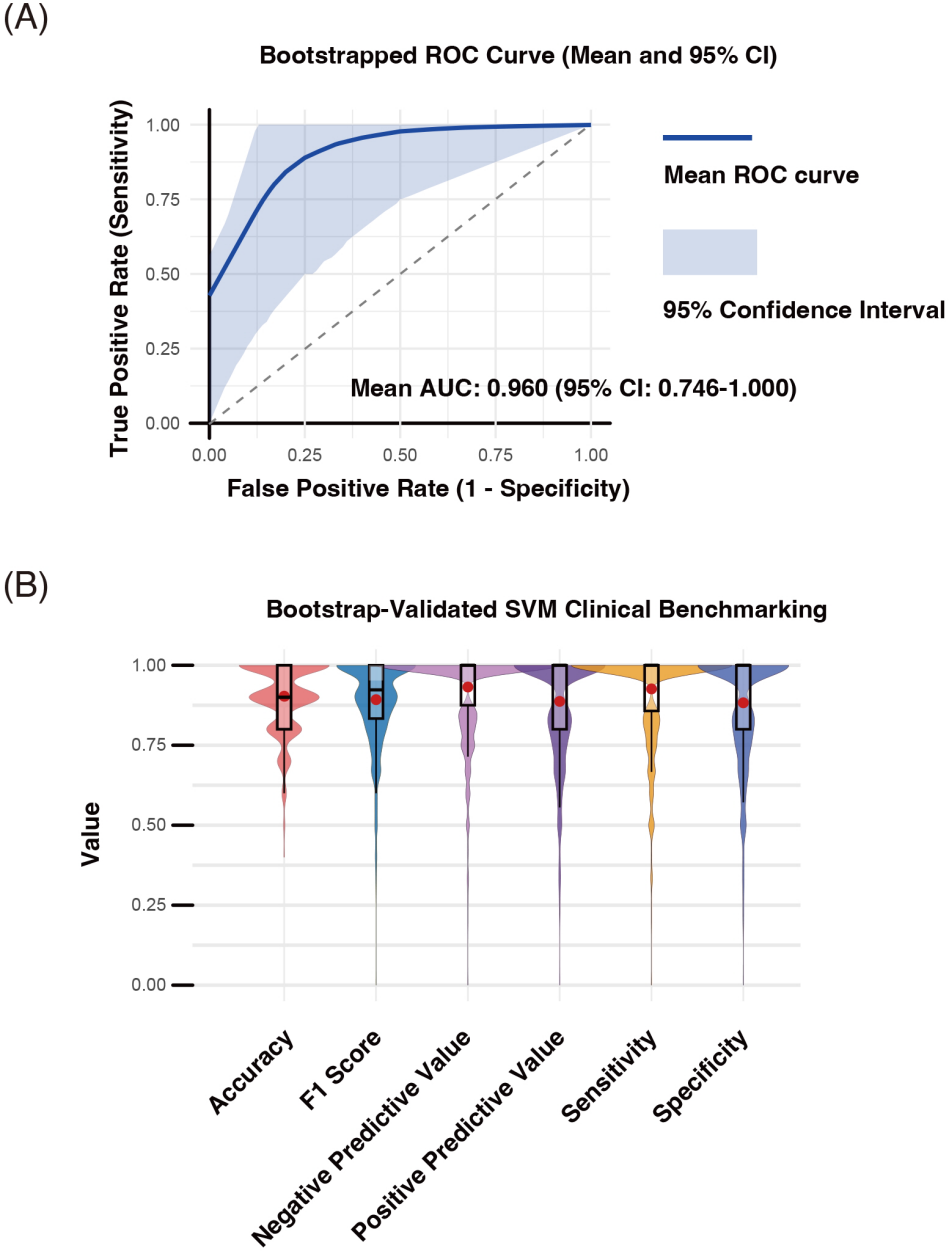
Internal performance assessment of discriminative T cell clusters (D-TCLs) for distinguishing seropositive and seronegative rheumatoid arthritis (SP-RA and SN-RA) using bootstrap-supported SVM modeling. Bootstrap validation (n = 1000 iterations) was performed by randomly dividing each sample into training and test sets, with SVM hyperparameters (cost and gamma) optimized via grid search. All validation and performance assessment were conducted internally; external validation using independent data remains necessary to fully establish generalizability. Performance metrics—including accuracy, area under the receiver operating characteristic curve (AUC-ROC), F1 score, negative predictive value (NPV), positive predictive value (PPV), sensitivity, and specificity—were computed for each bootstrap iteration. **(A)** Mean ROC curve and 95% confidence interval. The mean ROC curve (blue line) and its 95% confidence interval (shaded area) are shown (mean AUC-ROC = 0.960, 95% CI: 0.746–1.000). **(B)** Distribution of classification performance metrics. Violin and box plots summarize the distributions of accuracy, F1 score, sensitivity, specificity, PPV, and NPV across bootstrap samples; mean values are indicated by red dots. The results confirm the high discriminative power of D-TCLs, with an average accuracy of 86.2% (95% CI: 62%–100%), sensitivity of 85.7%, specificity of 80.9%, PPV of 82.3%, NPV of 87.4%, and F1 score of 0.823.

The mean ROC curve and its 95% confidence interval (mean AUC = 0.960, 95% CI: 0.746–1.000) are shown across all bootstrap iterations (**Figure 8A**).

The distributions of accuracy, F1 score, sensitivity, specificity, PPV, and NPV are summarized as violin and box plots, with mean values indicated by red dots (**Figure 8B**).

The average accuracy was 86.2% (95% CI: 62%–100%), sensitivity 85.7%, specificity 80.9%, PPV 82.3%, NPV 87.4%, and F1 score 0.823.

These results highlight the high discriminative power of D-TCLs as candidate immunophenotypic biomarkers for RA subgroup classification.

All validation was performed internally using bootstrap-supported train/test splitting; external validation with independent data remains essential to fully confirm generalizability.

The observed mean AUC of the SVM model using the true labels was 0.944. In the permutation test (n = 1000), the mean AUCs obtained with randomly shuffled labels were centered at 0.654 (range 0.608–0.879). The observed mean AUC was significantly higher than the entire permuted distribution (permutation test p-value = 0.0010), indicating that the predictive performance of the selected variables was highly unlikely to be attributable to chance alone. These results further support the robustness and true discriminative value of the D-TCLs for classifying ACPA status (**Supplementary Figure 10**).

The alternative classifier analysis confirmed that the predictive performance of the selected D-TCLs was not restricted to SVM. The mean AUCs for Elastic Net, Random Forest, and XGBoost models were 0.844 (95% CI: 0.500–1.000), 0.892 (0.650–1.000), and 0.835 (0.525–1.000), respectively (**Supplementary Figure 11**). These results demonstrate that the identified signature provides robust discrimination of RA subgroups across multiple machine learning algorithms.

## 4 Discussion

In this study, we identified six novel D-TCLs: TCL02, TCL21, TCL24, TCL31, TCL32, and TCL35, which can serve as biomarkers for distinguishing between SP-RA and SN-RA. Utilizing mass cytometry and machine learning, including the FlowSOM algorithm, we stratified patients by ACPA and RF status and characterized 44 distinct TCLs. This approach represents a significant advance, moving beyond traditional analyses that focus on memory and activated T cell subsets. In addition to these TCLs, our analysis of T cell subsets revealed critical immunological differences between SP-RA and SN-RA. Specifically, we observed a higher frequency of EM-CD4+ T cells and a reduced prevalence of Tregs in SP-RA compared to SN-RA. Moreover, the Tph/Treg ratio, significantly elevated in SP-RA, underscores the effector-dominant immune imbalance characteristic of this subtype. These findings suggest that the balance between effector and regulatory T cells, particularly Tph and Tregs, plays a pivotal role in distinguishing these RA subtypes and contributes to their distinct immunopathological profiles. Our comprehensive examination of the CD3+ T cell population, including naïve T cells, has broadened our understanding of T cell diversity, uncovering additional clusters and suggesting new potential biomarkers for RA. These findings not only enhance diagnostic precision but also deepen our understanding of RA pathogenesis.

Our study advances the understanding of CD4+ T cell heterogeneity in RA, particularly in distinguishing the immunological landscapes of SP-RA and SN-RA. Prior single-cell RNA sequencing studies have identified transcriptomic differences, such as splicing variations in PTPRC (CD45), critical for T cell activation, and CLEC2D, associated with lymphocyte counts and pro-inflammatory states, which may contribute to immune dysregulation in RA (35, 36). These molecular insights align with our findings of increased effector subsets, such as EM-CD4+ T cells, in SP-RA. ACPA titers, a hallmark of SP-RA, were positively correlated with EM-CD4+ and EM-CD8+ T cells and the Tph/Treg ratio, while inversely correlated with Tregs. These associations emphasize the effector-dominant immune response in SP-RA, driven by autoantibody production and inflammation. Additionally, CRP levels and disease activity indices (DAS28-CRP and SDAI) were strongly linked to Th17 and Th17.1 cells and the Th17/Treg ratio, reaffirming the role of Th17-mediated inflammation in RA (11, 14, 15).

Among CD4+ T cell subsets, activated Tph cells were significantly elevated in SP-RA, accompanied by a marked increase in the Tph/Treg ratio. This imbalance reflects an effector-dominant immune profile in SP-RA, where Tph cells drive B cell activation and autoantibody production (16, 17, 36). In contrast, SN-RA exhibited an increased prevalence of Tregs, which negatively correlated with ACPA titers. This suggests that Tregs play a protective role in SN-RA, mitigating inflammation and osteoimmunological damage (37, 38). Notably, clonal expansion of Tregs has been reported to be higher in ACCP- RA synovial fluid, potentially contributing to a more regulated immune environment in this subtype (36). To further explore the regulatory mechanisms underlying these observations, we analyzed the expression of co-stimulatory and inhibitory molecules within Treg subsets across RA subtypes. Notably, CTLA-4 expression in total Tregs and 4-1BB expression in naïve Tregs were significantly higher in SN-RA compared to SP-RA, suggesting a more functionally active regulatory phenotype in the seronegative subgroup. These molecular features align with the increased Treg frequency observed in SN-RA, and collectively point to a more robust immunoregulatory environment. In addition to suppressing effector T cells, Tregs—particularly those expressing CTLA-4—are also involved in modulating Tph-mediated B cell activation and autoantibody production. Therefore, these findings imply that the enhanced regulatory capacity in SN-RA may help restrain both cellular and humoral autoimmunity. In contrast, SP-RA appears to be characterized by reduced Treg quantity and function, contributing to an imbalance favoring effector responses. Together, these results highlight the functional heterogeneity of Tregs in RA and their potential role in shaping the distinct immunopathology of SP-RA and SN-RA.

Enhanced interactions between T cells and antigen-presenting cells, observed in ACCP- RA through ligand-receptor pairs such as CCR8-CCL18, may further support compensatory immune mechanisms in SN-RA (39). Metabolic differences in dendritic cells (DCs) may also contribute to these distinct immune profiles. Enhanced glycolysis in cDC2 has been shown to promote effector T cell activation, which could support the inflammatory phenotype of SP-RA, while a less inflammatory metabolic profile in pDCs may favor Treg expansion in SN-RA (40). This interplay between metabolic and immune regulation provides further insights into the mechanisms underlying RA subtypes and potential therapeutic avenues.

Among D-TCLs, TCL21 was notable. This cluster, distinguished by its CCR5+CXCR3+CCR6−HLA-DR+ profile, high PD-1 levels, with ICOS expression, and lacking CXCR5, is indicative of an activated Th1-type Tph-like cell (41). The relevance of this finding becomes apparent in SP-RA, characterized by autoantibody profiles akin to those found in systemic lupus erythematosus (41). Activated Th1-type Tph-like cells, represented by TCL21, are likely to play a significant role in SP-RA by modulating the inflammatory responses associated with the disease. This interpretation is supported by the correlation of TCL21 with DAS28-CRP and patient-reported VAS, linking it to disease activity and symptom severity. Our observations underscore the potential role of activated Th1-type Tph-like cells in SP-RA and contribute to the evolving understanding of activated Tph cells in autoimmune diseases.

Similarly, the characteristics of TCL02 as a CM CD4+ T cell highlight its potential role in sustaining long-term immune memory in RA, which is implicated in the maintenance of immunological memory to self-antigens, a core aspect of RA pathogenesis (42). This cluster is defined by the expression of markers such as CD38, ICOS, CD28, and CXCR3. The presence of these activation and costimulatory markers suggests that TCL02 cells are primed for rapid antigen responses, critical for continuous T cell activation and survival (43, 44). Although TCL02 is consistently detected in both SP-RA and SN-RA and shows increased prevalence in patients with RA compared with HCs, its utility as a biomarker for differentiating between these RA subgroups remains limited due to overlapping characteristics. Nevertheless, the ubiquitous presence of TCL02 across these patient groups underscores its potential as a therapeutic target throughout the RA spectrum.

The identification of two CD4−CD8− double-negative T cell clusters, TCL31 and TCL35, predominantly in SP-RA and characterized by CD161+ and HLA-DR+CD38+TIM-3+ expression respectively, offers critical insights into their diverse immunological roles in SP-RA. TCL31, newly recognized and marked by CD161+ expression, may influence SP-RA pathology. Recent research has highlighted the role of CD161+ γδ T cells, which are key in inflammatory responses because they secrete IFN-γ and IL-17, in the pathogenesis of chronic pulmonary disorders, such as bronchiectasis (45). Similarly, TCL31 appears to mirror the immunological phenotype of CD161+ γδ T cells. Notably, our supplementary flow cytometry analysis revealed that γδ T cells comprise approximately 50% of the CD4^-^CD8^-^ double-negative T cells in RA peripheral blood (**Supplementary Figure 4**), supporting the possibility that TCL31 reflects a γδ T cell–enriched population within this subset. In RA, the production of citrullinated proteins and the consequent activation of ACPA extend beyond the synovium to the lungs (5, 46), linking to an increased incidence of bronchial abnormalities in patients with SP-RA (46). This systemic manifestation, in addition to the expression of CD161 on natural killer T cells and mucosal-associated invariant T cells, which are activated and reduced in the peripheral blood of patients with RA (47, 48), similar to TCL31, highlights the need for experimentally confirming the role of CD161 in CD4−CD8− double-negative T cells in the inflammatory pathways of SP-RA. TCL35, characterized by HLA-DR+CD38+TIM-3+ expression, exhibits multifaceted functionality. The presence of HLA-DR and CD38, markers associated with T cell activation and antigen presentation, combined with TIM-3, recognized for its regulatory and suppressive functions, suggests a dual role for TCL35 in SP-RA, potentially contributing to both the exacerbation and regulation of the autoimmune response. Unraveling the functions of these clusters could pave the way for novel targeted therapeutic strategies in SP-RA, ultimately enhancing treatment efficacy and improving patient outcomes. These observations underscore the need for further investigation to directly establish the mechanistic contributions of these TCLs to RA pathophysiology.

As a next step, validating whether D-TCLs are present and functionally relevant in RA target tissues such as the synovium or lung will be important. However, directly matching CyTOF-defined clusters in blood with scRNA-seq-defined populations in tissue is technically challenging due to differences in data modality and tissue-specific immune states. Future studies using matched samples for CyTOF and scRNA-seq may help bridge this gap, allowing the identification of peripheral blood phenotypes that reflect tissue-resident pathogenic T cells. This integrative approach could also facilitate the development of blood-based biomarkers and clarify links between D-TCLs and clinically important features such as treatment resistance and extra-articular manifestations.

The identification of T cell clusters like TCL24 and TCL32, marked by low phenotype expression, highlights an intriguing aspect of the RA immune landscape. Although these cells display minimal active markers, their notable presence across RA subtypes invites deeper examination. It is unclear whether these clusters have direct immunological functions or are simply variations seen in RA. Given their intriguing presence, it is crucial to investigate these clusters to understand their potential impact on RA pathogenesis. Until such investigations are conducted, the functional roles of TCL24 and TCL32 should be considered undetermined.

The identification of potential cellular biomarkers in SN-RA, specifically TCL10 and TCL29, is significant. TCL10, characterized by a cluster of activated CD4+ T cells, showed increased expression of chemokine receptors crucial for T cell activation and trafficking, underscoring its role in the pathogenesis of SN-RA. Conversely, TCL29, composed of naïve CD4+ T cells with atypical activation markers and chemokine receptors, indicates an aberrant activation state. This distinction highlights the need for further investigation into how this cluster contributes to the distinctive immunopathology observed in SN-RA, potentially affecting disease progression and therapeutic responses.

Although FSM-ATCL-DS did not qualify for inclusion in D-TCL analysis, some ATCLs, such as ATCL06, exhibited notable patterns relevant to RA. ATCL06, more prevalent in SP-RA than in SN-RA (**Supplementary Figure 6B**), mirrored the phenotype of activated Th1-type Tph-like cells, characterized by CXCR3+, CCR5+, and CCR6−, similar to TCL21 (**Supplementary Figure 5C**). This similarity suggests a role in the pathogenesis of SP-RA, reinforcing prior research on the involvement of activated Tph cells in RA (16, 49), and may have implications in therapeutic strategies. ATCL03, which exhibited a phenotype consistent with activated Treg cells (CD4^+^CD25^+^CD127^low), tended to be more abundant in SN-RA than in SP-RA, although the difference was not statistically significant. This lack of significance may reflect the modest effect size or biological variability within subgroups. It also underscores a limitation of surface marker–based unsupervised clustering, which, while effective for exploratory phenotyping, may not fully resolve functionally distinct subsets like activated Tregs when compared to manual gating based on well-established definitions.

The identification of D-TCLs in RA using FlowSOM revealed complex patterns that surpass traditional analysis capabilities, demonstrating the significant potential of machine learning in immunological research. Our study focused on the single-cell analysis of 25 surface antigens; however, it is crucial to recognize that the phenotypic complexity of immune cells far exceeds this scope. FlowSOM, an unsupervised machine-learning technique, has excelled in differentiating between SP-RA and SN-RA T cell clusters, outperforming conventional methods by achieving higher accuracy. This success not only paves new pathways in immunology research, including the discovery of novel biomarkers and the exploration of pathophysiological mechanisms, but also underscores the need for broader investigations. Future research should aim to validate these findings in independent datasets to enhance their clinical applicability and build on our discoveries.

A primary limitation of our study lies in the reliance on surface marker expression—particularly chemokine receptor-based definitions—for characterizing conventional T cell subsets such as Th and Treg. While these phenotypic definitions are grounded in widely accepted immunological criteria, they may not fully capture the underlying functional or transcriptional heterogeneity within these subsets. For example, cytokine co-expressing T cells such as IFN-γ^+^ IL-17^+^ CD4^+^ T cells, which are increasingly recognized as pathogenic in RA and other autoimmune diseases (50), cannot be reliably distinguished from conventional Th1 or Th17 cells using surface markers alone. Although Th17.1 cells (CXCR3^+^CCR6^+^), included in our analysis, are considered to partially reflect this hybrid population (51), surface phenotype alone may not capture their full functional diversity. In contrast, the clustering of TCLs and ATCLs was based on a broader panel of surface markers; however, this unsupervised approach, diverging from traditional manual gating analyses of T cell subsets, may not fully capture T cell function or differentiation, potentially missing crucial immunological insights. However, the unsupervised phenotypic clustering approach was invaluable in identifying distinct D-TCLs. Subsequent functional estimation of these clusters, informed by established immunological knowledge, revealed their potential roles. However, clusters such as TCL24 and TCL32 that showed minimal marker expression, posed challenges in interpreting their role in disease pathology. Conversely, the analysis of clusters such as TCL21 and TCL34 was informative, enhancing our understanding of this phase of the study. By integrating established immunological insights with our phenotypic clustering, we addressed some inherent limitations of our methodology and facilitated detailed analyses that were uniquely possible through this approach.

A further limitation is the potential variability introduced by differences in data distribution between Mass Cytometry and Flow Cytometry. While we carefully adjusted gating strategies and positivity thresholds to account for these differences, subtle variability in data distribution may still affect the precision of subset identification. This highlights the inherent challenges in reconciling data generated by different platforms, despite rigorous methodological efforts.

Another significant limitation is the relatively small patient sample size, which may impact the statistical power and reliability of the results. Although efforts were made to adjust for differences in patient backgrounds using IPW, further validation in a larger, more closely-matched patient cohort is essential. Additionally, the lack of external validation using independent datasets limits the generalizability of our findings. Although our T cell clusters demonstrated robustness in SVM analysis with extensive bootstrap support (n = 1000), achieving impressive accuracy, these results were only validated within our initial patient cohort. It is critical for future studies to validate these T cell phenotypes in independent cohorts to establish their clinical applicability and confirm their role in RA pathophysiology and treatment. This study serves as an important step in identifying target cell populations for future large-scale validation, emphasizing the foundational value of our findings in advancing research into the pathophysiology of RA.

In conclusion, this exploratory study identified significant differences in T cell phenotypes between SP-RA and SN-RA. These distinctions suggest their potential as biomarkers for autoantibody production and response to altered self-antigens. In addition to offering insights into factors influencing joint prognosis and extra-articular complications in SP-RA, these phenotypic variations contribute to a deeper understanding of the immunological complexities in RA heterogeneity. Our findings increase the understanding of the intricate mechanisms of RA and lay the foundation for future investigations into the disease’s cellular biology. It paves the way for developing more targeted therapeutic strategies tailored to the nuanced needs of individual patients with RA.

## Data Availability

Due to the complex nature of the data used in this study, the datasets are not publicly available. However, the data that support the findings of this study are available from the corresponding author upon reasonable request. Interested researchers should contact the corresponding author to obtain access to the data, subject to necessary approvals and in compliance with applicable data protection laws. Requests can be sent to snb51961@med.nagoya-cu.ac.jp.

## Glossary

RA: Rheumatoid arthritis
HCs: Healthy controls
SP-RA: Seropositive RA
SN-RA: Seronegative RA
PBMC: Peripheral blood mononuclear cell
ACPA: Anticyclic citrullinated peptide antibody
CRP: C-reactive protein
MMP-3: Matrix metalloproteinase-3
RF: Rheumatoid factor
DAS28-CRP: Disease activity score 28-joint count C-reactive protein
SDAI: Simplified disease activity index
NSAID: Nonsteroidal anti-inflammatory drug
CM: Central memory
EM: Effector memory
TEMRA: Terminally Differentiated Effector Memory T cells Re-expressing CD45RA
CD4-SP: CD4 single positive
CD8-SP: CD8 single positive
IL: Interleukin
HLA-DR: Human leukocyte antigen-DR isotype
Th cell: T helper cell
Treg: Regulatory T cell
Tph: Peripheral helper T cells
CTLA-4: Cytotoxic T-lymphocyte-associated antigen-4
PD-1: Programmed death-1
LAG-3: Lymphocyte activation gene 3
ICOS: Inducible T cell costimulator
TIM-3: T cell immunoglobulin and mucin domain-containing protein 3
t-SNE: t-Distributed stochastic neighbor embedding
TCL: T cell cluster
ATCL: Activated T cell cluster
LOOCV: Leave-one-out cross-validation
IPW: Inverse probability weighting
adaptive LASSO: Adaptive least absolute shrinkage and selection operator
gating-TCS-DS: Gating T cell subset dataset
FSM-TCL-DS: FlowSOM T cell cluster dataset
FSM-ATCL-DS: FlowSOM activated T cell cluster dataset
