# Supplementary material for: Comprehensive and advanced T cell cluster analysis for discriminating seropositive and seronegative rheumatoid arthritis
Source: Front Immunol. 2025 Jul 24;16:1491041. doi: 10.3389/fimmu.2025.1491041 (PMC12328428; doi:10.3389/fimmu.2025.1491041)
Supplement: Supplementary file 1 [file DataSheet1.pdf]

Supplementary Material

1 Supplementary Figures and Tables

1.1 Supplementary Figures

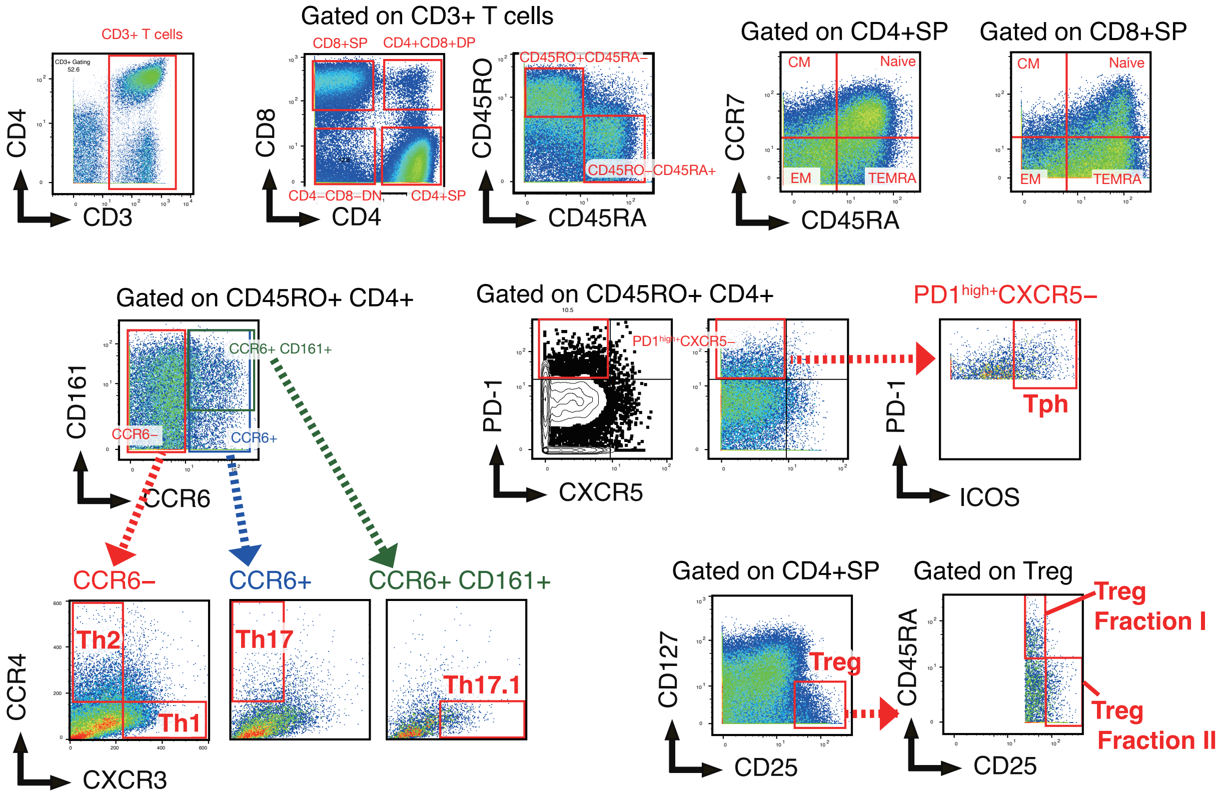

Supplementary Figure 1. Manual gating strategy for T cell subsets in mass cytometry.

Manual gating strategy used to analyze T cell subsets identified using mass cytometry. Based on established markers, the figure delineates sequential gates applied to CD3+ T cell population to categorize subsets of Th1, Th2, Th17, Th17.1, Tph, and Treg (fractions I and II) as well as naïve, terminally differentiated effector memory T cells re-expressing CD45RA (TEMRA), central memory, and effector memory T cells. Gating criteria included markers such as CD4, CD8, CD25, CD127, CD45RA, CD45RO, CCR7, CXCR3, CCR4, CCR6, CXCR5, PD-1, ICOS, and CD161. Each panel displays a step in the gating hierarchy, initiating with broad classifications and progressively refining to specific cell types.

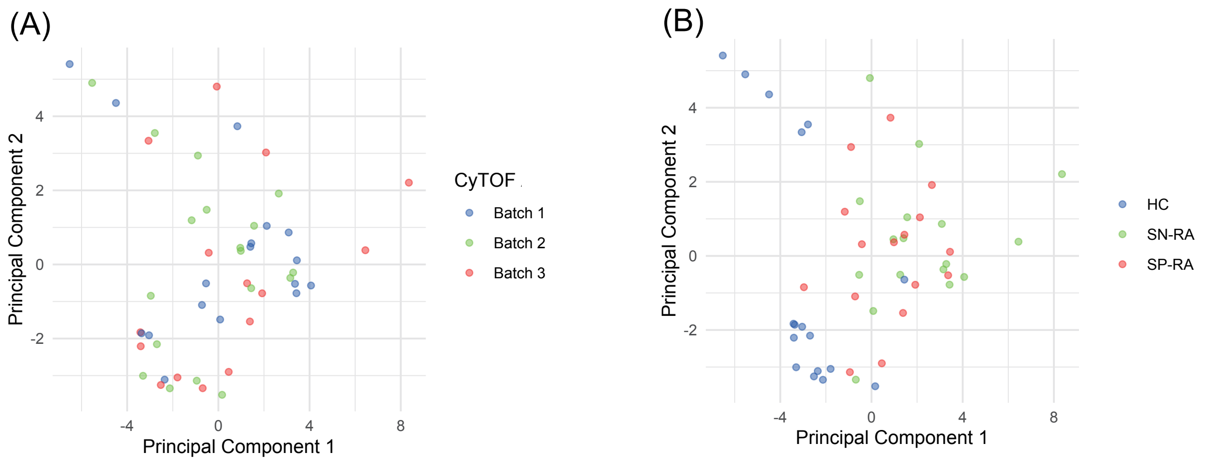

Supplementary Figure 2. Principal Component Analysis of normalized CyTOF data to assess technical and biological variation.

(A) PCA plot colored by CyTOF acquisition batch. No apparent clustering was observed by batch, indicating that normalization effectively mitigated inter-run variability.

(B) PCA plot colored by immunological group (SP-RA, SN-RA, HC). Healthy controls exhibited relatively tight clustering, whereas RA samples showed broader dispersion, reflecting expected immunological heterogeneity.

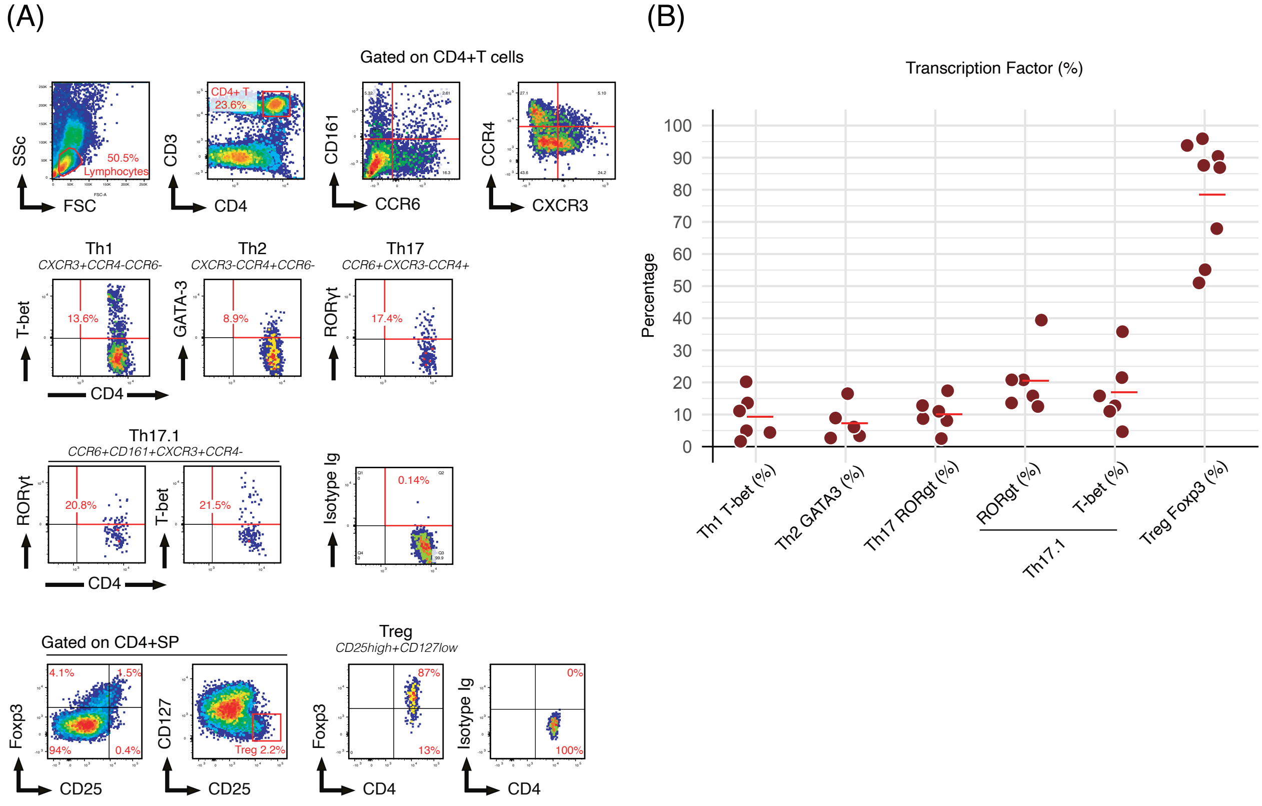

Supplementary Figure 3. Validation of transcription factor expression in surface marker-defined CD4⁺ T cell subsets.

(A) Representative flow cytometry plots showing intracellular expression of lineage-defining transcription factors in surface marker-defined CD4⁺ T cell subsets from RA patients. Th1 cells were assessed for T-bet expression, Th2 for GATA3, Th17 and Th17.1 for RORγt, and Treg cells for Foxp3. Gating strategies were based on established chemokine receptor and surface marker combinations.

(B) Summary graphs displaying the proportion of transcription factor-positive cells within each surface marker-defined subset. Foxp3 expression was analyzed in n = 8 RA samples (mean 78.6% ± 16.8%), while T-bet, GATA3, and RORγt were analyzed in n = 6. The mean percentages (± SD) for each subset were as follows: Th1 (T-bet): 9.3% ± 6.3%, Th2 (GATA3): 7.3% ± 4.6%, Th17 (RORγt): 10.1% ± 4.6%, Th17.1 (RORγt): 20.5% ± 9.0%, Th17.1 (T-bet): 16.9% ± 9.8%, Treg (Foxp3): 78.6%

± 16.8%. Red horizontal bars indicate mean values. The data illustrate high concordance between surface phenotype and transcription factor expression in Tregs, and modest expression in Th subsets, indicating phenotypic-functional heterogeneity.

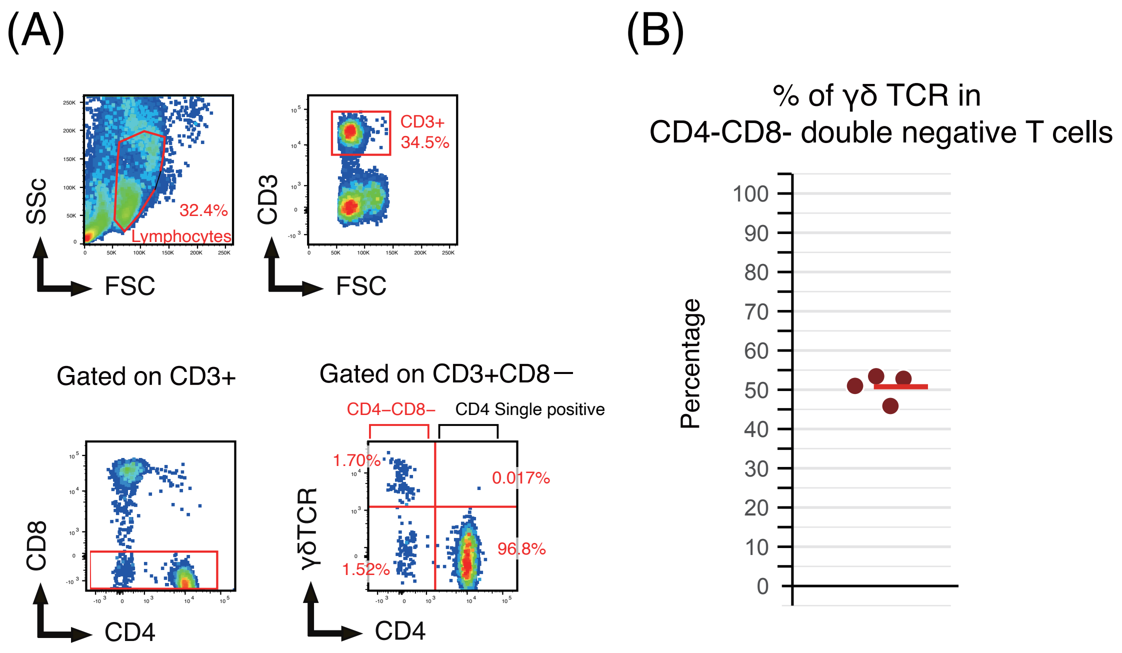

Supplementary Figure 4. γδ T cell composition within the CD4⁻CD8⁻ double-negative T cell population in RA.

(A) Representative flow cytometry plots showing the distribution of γδ T cells within $CD3^+CD8^-$ T cells from RA peripheral blood. Cells were first gated on $CD3^+CD8^-$ lymphocytes, and then subdivided into $CD4^+$ single-positive (CD4 SP) and $CD4^-CD8^-$ double-negative (DN) subsets. γδTCR expression was subsequently analyzed within each subset. This analysis revealed that γδ T cells are largely confined to the DN population, with minimal presence in CD4 SP T cells.
(B) Summary graph showing the frequency of $γδTCR^+$ cells among $CD4^-CD8^-$ double-negative T cells in four RA patients. Red horizontal bars indicate the mean. These results demonstrate that γδ T cells represent a substantial proportion of the DN T cell compartment in RA peripheral blood.

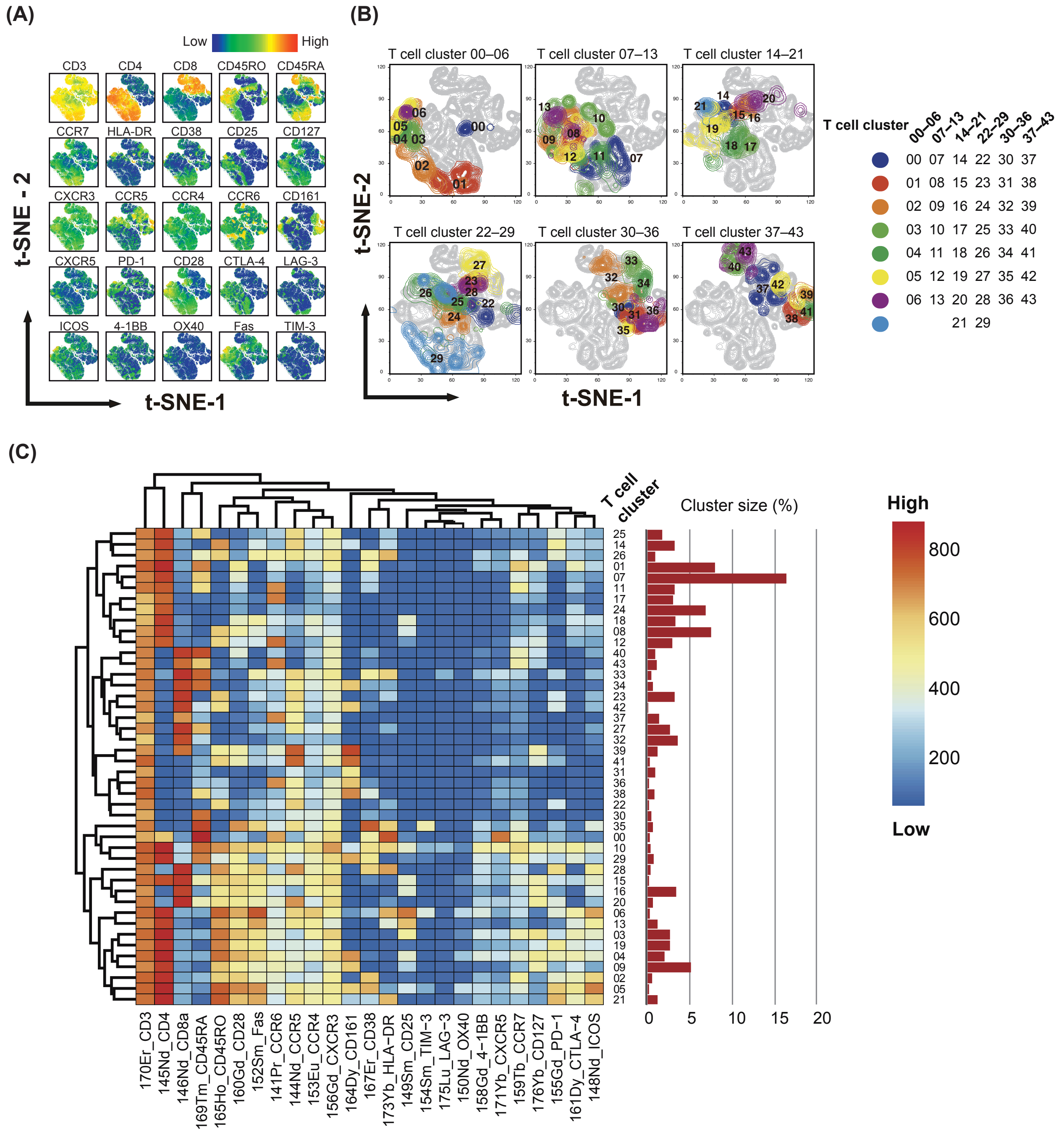

Supplementary Figure 5. High-dimensional T cell clustering and phenotypic characterization using FlowSOM and t-SNE.

(A) High-dimensional data from 25 T cell markers collected from 50 participants were analyzed using t-distributed stochastic neighbor embedding (t-SNE) to visualize the data in two dimensions. CD3+ T cells were clustered using the FlowSOM algorithm, which employs self-organizing maps to identify patterns within high-dimensional cytometry data. This resulted in the detection of 44 distinct

T cell clusters (TCLs, cluster numbers 00–43). The expression levels of the 25 cell surface markers across these clusters are overlaid on the t-SNE plot as a heatmap.

(B) The t-SNE projection of all clusters is shown as a contour plot, with numeric cluster IDs (e.g., 00, 01, etc.) displayed directly on the plot. For clarity, the corresponding cluster colors and IDs are also presented in a legend to the right of each plot. To maximize visual differentiation, clusters were grouped and plotted across several panels, with each panel using a distinct color palette to avoid color repetition.

(C) The immunophenotypic features of the 44 TCLs are detailed through a comprehensive heatmap displaying the expression of the 25 surface markers for each cluster. Adjacent to this, a red bar graph represents the percentage each TCL comprises within the overall concatenated dataset, providing a quantitative measure of each cluster's prevalence.

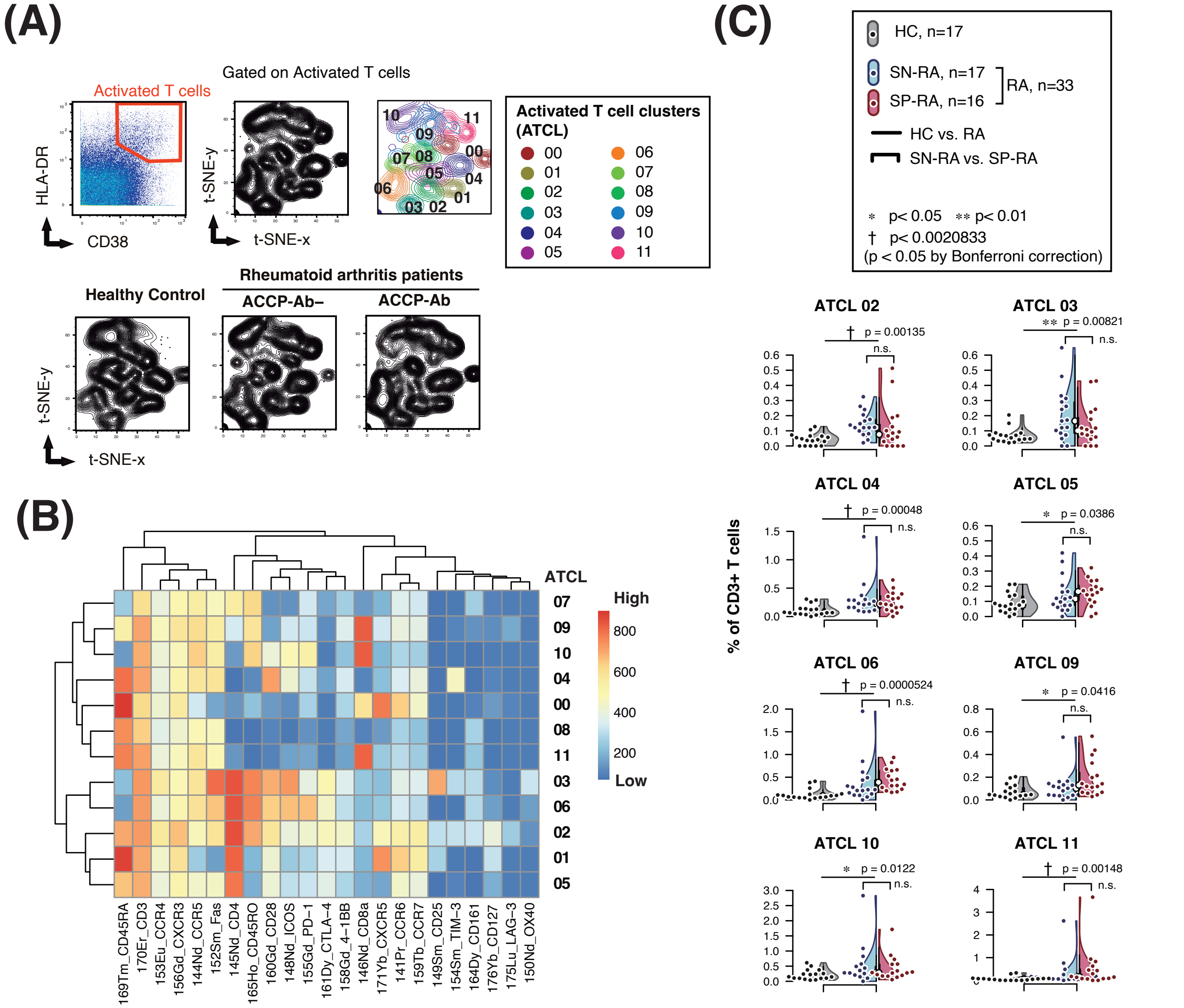

Supplementary Figure 6. Delineation and immunophenotypic profiling of activated T cell clusters in rheumatoid arthritis. (**A**) The panel displays cytometry plots using a pseudocolor plot to gate activated (HLA-DR+CD38+) T cells within the CD3+ population. The gated cells were visualized on a two-dimensional t-SNE map, represented through a contour plot that illustrates the density and distribution of these cells. Subsequently, FlowSOM analysis identified 12 distinct activated T cell clusters (ATCL00–11). Each cluster is uniquely colored on the t-SNE map to facilitate differentiation, with the contour plot format emphasizing the distinct regions occupied by each cluster. (**B**) The immunophenotypic characteristics of the 12 ATCLs are presented in a heatmap, depicting the expression profiles for 23 surface markers, excluding HLA-DR and CD38. (**C**) Proportional distribution of eight representative ATCLs (among 12 total) within $CD3^{+}$ T cells, stratified by group: healthy controls (HC), seronegative RA (SN-RA), and seropositive RA (SP-RA). Only clusters with statistically significant differences in at least one group comparison are shown. Statistical analyses were conducted using the Mann–Whitney U test. *$p < 0.05$; **$p < 0.01$; †$p < 0.0020833$ (Bonferroni-corrected significance threshold for 24 comparisons).

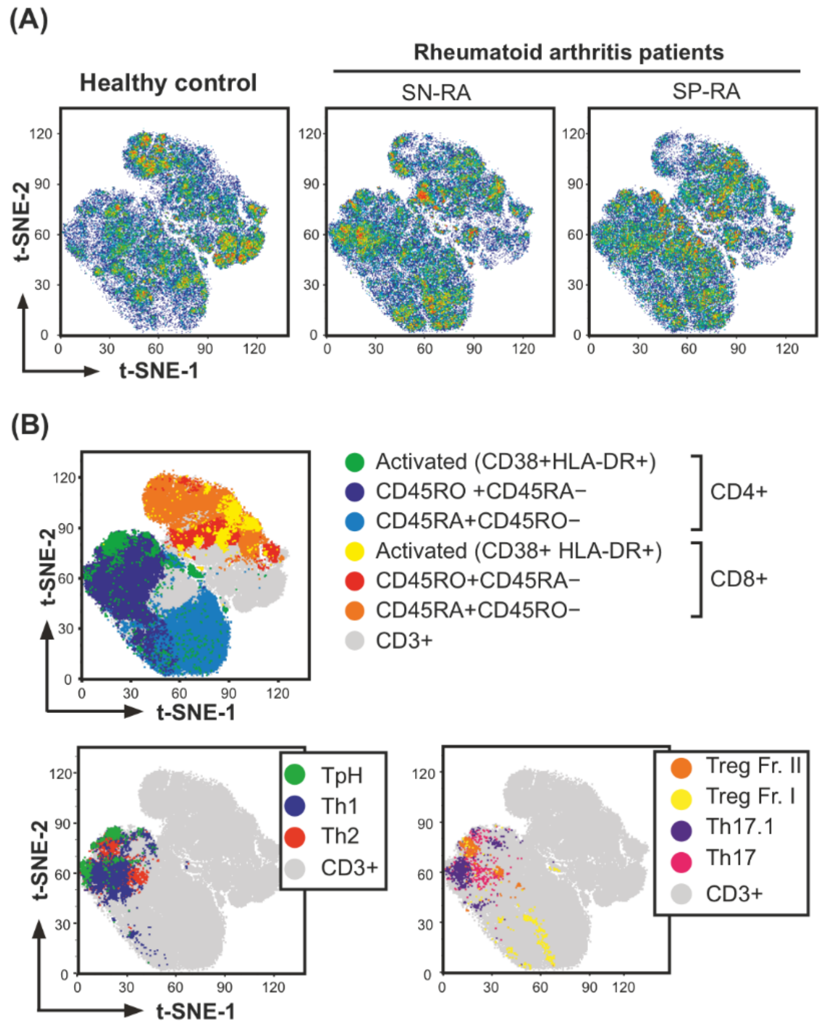

Supplementary Figure 7. Visualizing T cell subsets and distributions in rheumatoid arthritis and healthy controls using t-SNE. (**A**) T cell marker data from 50 participants were processed through t-SNE for two-dimensional visual mapping. The panel displays the t-SNE plot featuring pseudocolor plots that show variations in cell density, delineating differences in T cell distribution across the healthy controls (HCs), seropositive rheumatoid arthritis (SP-RA), and seronegative rheumatoid arthritis (SN-RA) groups. (**B**) Overlaid on a collective t-SNE map of all patient data are specific T cell subsets, each marked with a distinct color. These overlays demonstrate the relative abundance and distribution patterns of these T cell subsets.

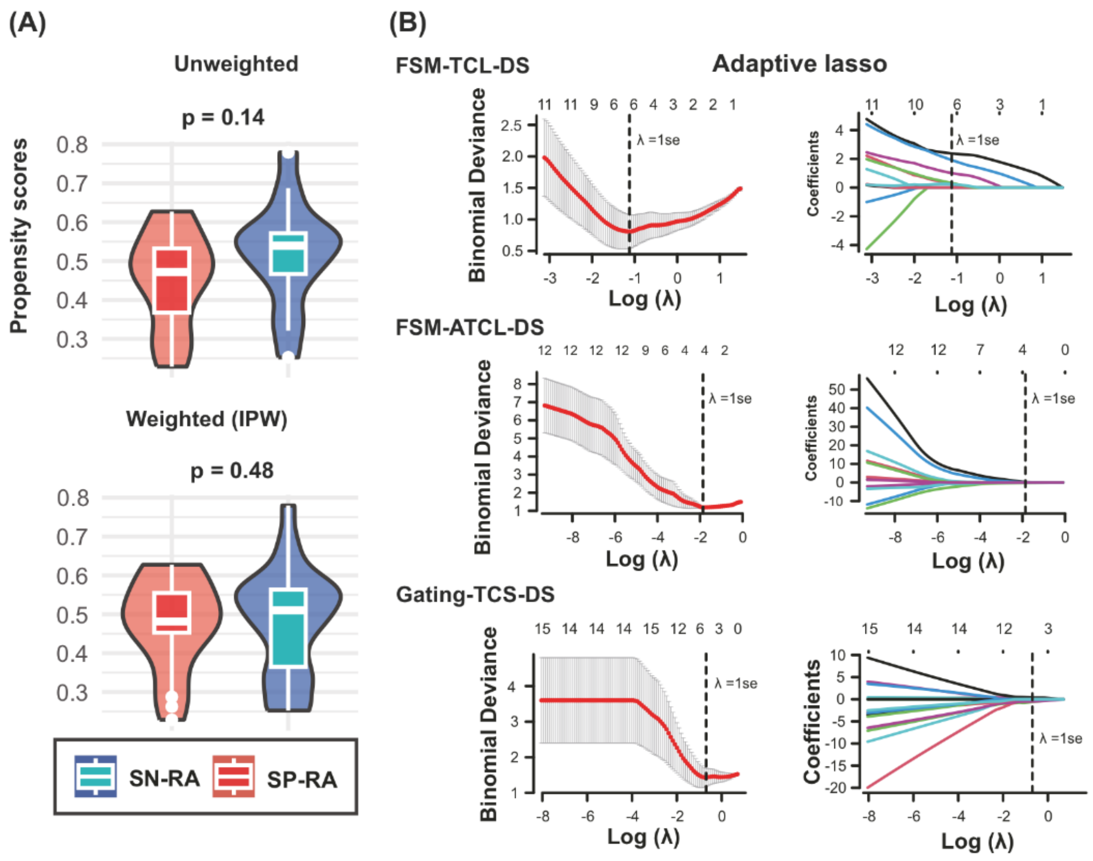

Supplementary Figure 8. Examination of IPW adjustments and regularization paths in adaptive LASSO analysis. (**A**) Propensity score adjustment comparison. The panel illustrates the distribution of propensity scores for seronegative rheumatoid arthritis (SN-RA) and seropositive rheumatoid arthritis (SP-RA) groups before and after adjustments using inverse probability weighting (IPW). IPW adjustments were made using patient background variables, such as sex, age, symptom duration, NSAID usage, and DAS28-CRP, to control for confounding factors. Unweighted (left) and weighted (right) violin and box plots compare these scores across the two groups, emphasizing the effectiveness of IPW in achieving balance. Statistical significance was assessed using the Mann–Whitney *U* test, with adjustments for the weights in the IPW version, and *p*-values are presented to indicate differences in score distribution. (**B**) Regularization paths in adaptive LASSO. Panel displays the regularization paths for adaptive LASSO analysis across three datasets: FSM-TCL-DS, FSM-ATCL-DS, and gating-TCS-DS. Each plot shows how the coefficients of variables shrink as the regularization parameter increases, detailing the process of variable selection crucial for distinguishing between RA subtypes. Selection of the final model is guided by the 1-standard error (1se) rule, which is employed to balance predictive accuracy against overfitting. This method is highlighted by a vertical dashed line in each plot, indicating the choice of a simpler model that enhances robustness and minimizes overfitting by selecting variables within one standard error of the best-performing model.

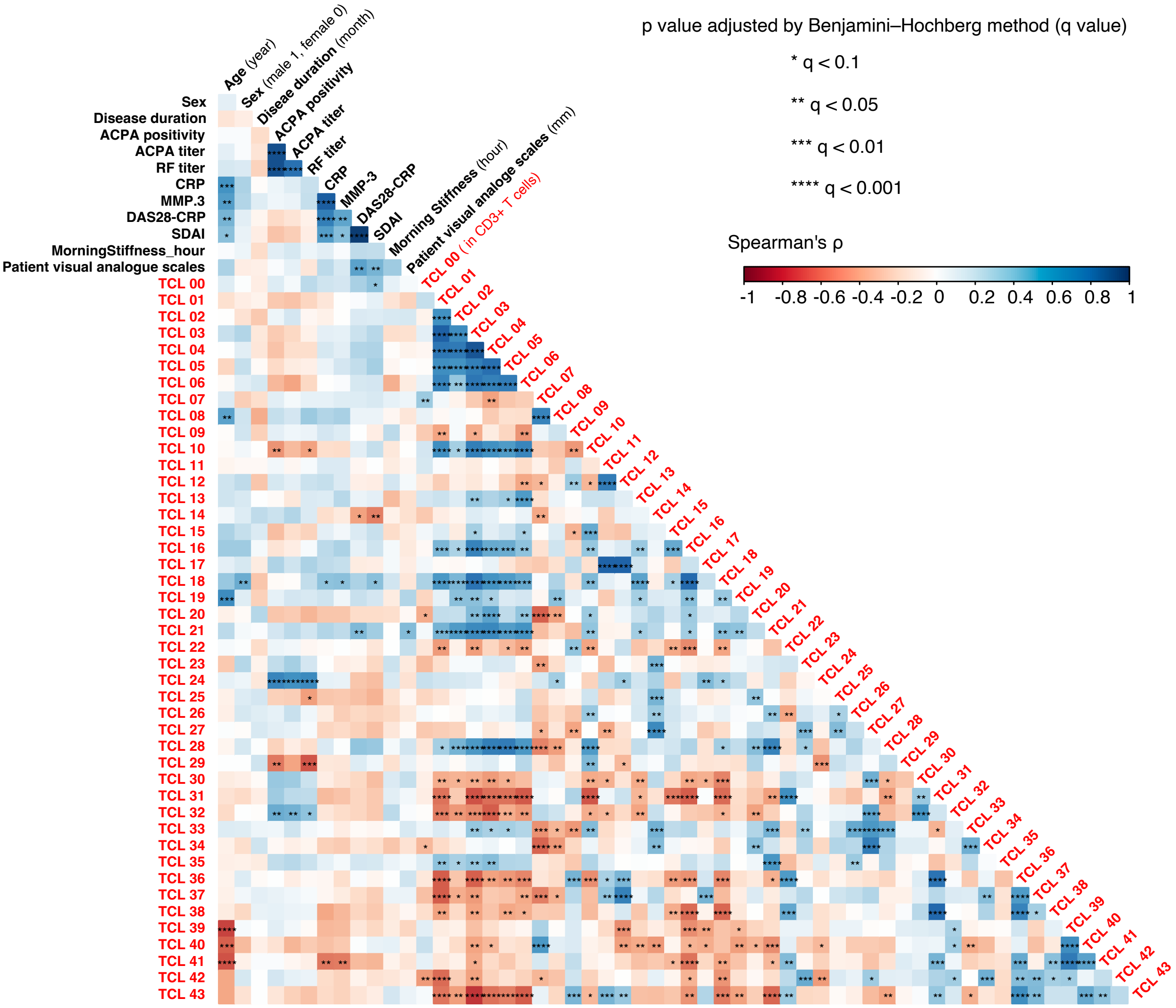

Supplementary Figure 9. Comprehensive correlation analysis across 44 T cell clusters (TCLs) and clinical characteristics in patients with rheumatoid arthritis (n = 33). Correlation coefficient matrix plot illustrates the relationships between 44 T cell clusters (TCLs) and clinical characteristics in patients with rheumatoid arthritis, represented using Spearman's correlation coefficient (ρ). Each cell in the heatmap is colored according to the correlation strength, on a scale of −1 (strong negative) to +1 (strong positive), denoting increasing correlation strength. *P*-values were adjusted for multiple comparisons using the Benjamini–Hochberg method. Adjusted *q*-values are annotated within the matrix: $^{*}q < 0.1$, $^{**}q < 0.05$, $^{***}q < 0.01$, and $^{****}q < 0.001$, highlighting levels of statistical significance.

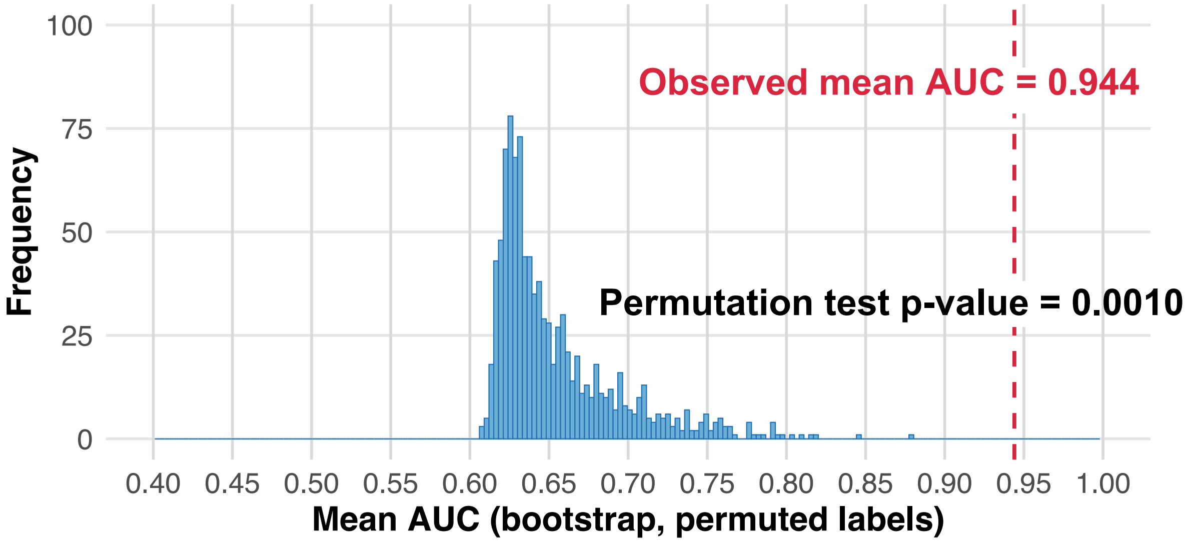

Supplementary Figure 10. Permutation Test for SVM-Based AUC Performance
Distribution of mean AUC values obtained by permutation testing (n = 1000) using SVM with fixed hyperparameters. The histogram shows the distribution of mean AUCs for SVM models trained on datasets with randomly shuffled ACPA status labels (teal bars). The red dashed vertical line indicates the observed mean AUC (0.944) obtained with the true labels. The observed mean AUC was significantly higher than the permuted distribution (permutation test p-value = 0.0010), supporting the true predictive value of the selected variables.

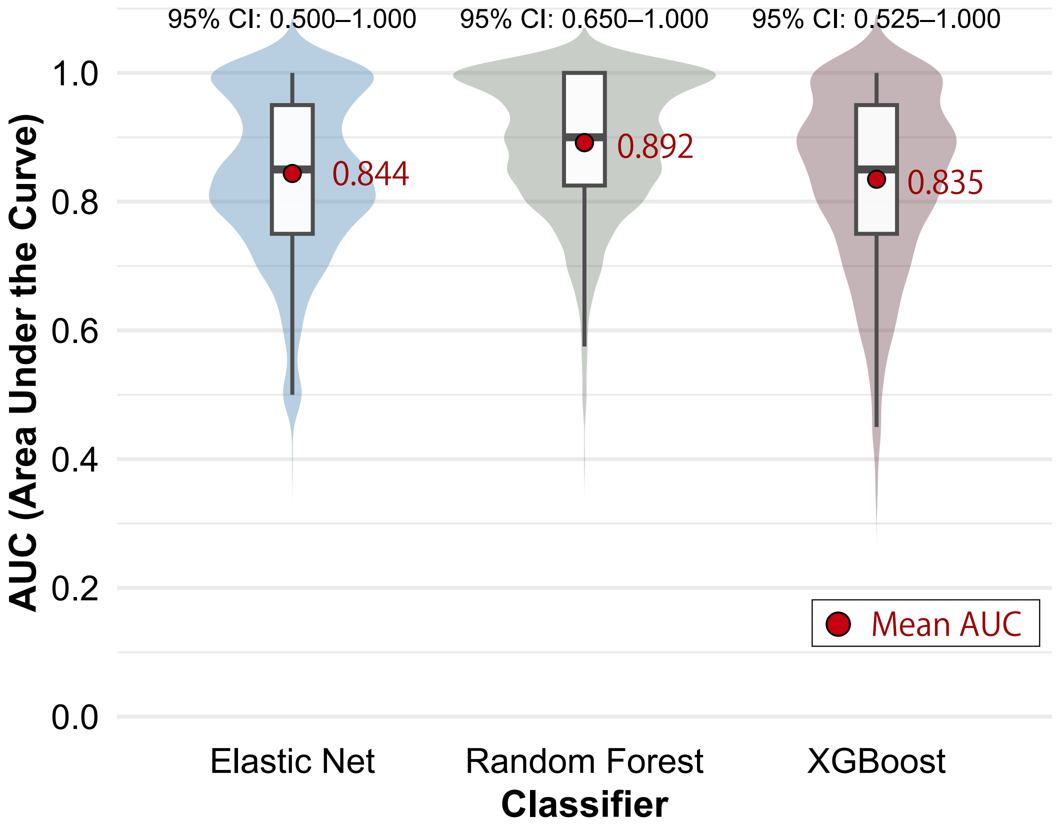

Supplementary Figure 11. Comparison of Predictive Performance Across Classifiers

Distribution of AUC values for Elastic Net, Random Forest, and XGBoost models trained and evaluated using the selected D-TCLs and bootstrap resampling (n = 1000). Violin and box plots show the distribution and interquartile range for each classifier. Mean AUC and 95% confidence intervals are indicated above each group. All classifiers achieved similarly high AUCs, indicating the robustness of the predictive signature regardless of the choice of machine learning algorithm.

1.2 Supplementary Table

Supplementary Table. Antibodies conjugated with metal isotopes for T cell phenotyping in mass cytometry.

Metal isotope-conjugated antibodies utilized for the phenotyping of peripheral blood T cells. Details of 25 distinct antibodies, specifying the metal isotope label, target molecule, and clone name for each, are listed.

label	target	clone
149Sm	CD25 (IL-2R)	2A3
141Pr	CD196 (CCR6)	11A9
145Nd	CD4	RPA-T4
153Eu	CD194/CCR4	L291H4
156Gd	CD183 (CXCR3)	G025H7
165Ho	CD45RO	UCHL1
146Nd	CD8a	RPA-T8
164Dy	CD161	HP-3G10
170Er	CD3	UCHT1
176Yb	CD127 (IL-7Ra)	A019D5
144Nd	CD195 (CCR5)	NP-6G4
159Tb	CD197 (CCR7)	G043H7
169Tm	CD45RA	HI100
173Yb	HLA-DR	L243
167Er	CD38	HIT2
160Gd	CD28	CD28.2
150Nd	CD134 (OX40)	ACT35
152Sm	CD95/Fas	DX2
154Sm	TIM-3	F38-2E2
155Gd	CD279 (PD-1)	EH12.2H7
161Dy	CD152 (CTLA-4)	14D3
148Nd	CD278/ICOS	C398.4A
158Gd	CD137/4-1BB	4B4-1
175Lu	CD223/LAG-3	11C3C65
171Yb	CD185/CXCR5	51505

2 Supplementary Methods

This document provides additional details and clarifications for the methodologies described in the Main Methods section of the manuscript. Each section in the Supplementary Methods corresponds directly to the respective section in the Main Methods (e.g., Supplementary Methods 2.2 corresponds to Main Methods Section 2.2). This structure ensures that readers can easily locate supplementary information relevant to specific methodological details.

2.2 Data Adjustment and Validation of Cytometry Analysis

To ensure the accuracy of our analysis, we adopted specific strategies to account for differences between flow cytometry and mass cytometry data. For mass cytometry data, axis settings and positivity thresholds were carefully adjusted to reflect differences in data distribution compared to flow cytometry. In cases where population boundaries were unclear, we referred to positivity rates obtained from parallel analyses using flow cytometry for the same samples. These adjustments ensured that the results were interpretable and aligned with established cell subset definitions.

2.3 Unsupervised FlowSOM Clustering of T Cell and Activated T Cell Clusters in RA Using Mass Cytometry

2.3.1 Preparatory Steps

CD3+ T cells were gated from CyTOF data following these steps:

- Residual bead removal: Using 140Ce_beads.
- Doublet exclusion: Based on Event length and 191Ir.
- Dead cell elimination: Using 191Ir and 198Pt.

2.3.2 FlowSOM Clustering

FlowSOM clustering was applied to concatenated CyTOF data gated on CD3+ cells from 50 participants, including 17 HCs. The inclusion of control samples provided a baseline for identifying both normal and pathological T cell clusters.
Key parameters and steps included:

- **Initial clustering:** Generated 200 clusters (nodes) using self-organizing maps.
- **Metacluster refinement:** The initial 200 clusters were hierarchically merged using dendrogram analysis. The final metacluster number (44) was selected a priori, based on empirical inspection of dendrogram structure, aiming to preserve major biological populations (e.g., approximately 20 clusters each for CD4+ and CD8+ lineages) and to avoid merging distinct immunophenotypes. This cluster number provided the optimal balance between interpretability and resolution.

- **Activated T cell analysis:** For the CD3+HLA-DR+CD38+ subset, 100 initial clusters were generated using FlowSOM, which were then hierarchically merged to obtain 12 activated T cell clusters (ATCL00–11).

- **Rationale for fixed cluster number:** Alternative cluster numbers were not systematically tested in order to avoid overfitting and to prevent post hoc (data-driven) selection of the cluster number. All subsequent analyses used this pre-specified 44-cluster structure.

2.3.3 Visualization and Data Analysis

- t-SNE visualization: Used for high-dimensional data visualization.
- Heatmaps: Created to depict surface marker expression across TCLs and ATCLs.
- Datasets established:
  i. FSM-TCL-DS: Derived from T cell cluster analysis within the CD3+ T cell region.
  ii. FSM-ATCL-DS: Focused on ATCLs within the activated T cell region.

2.3.4 Batch Effect Evaluation and Sample Distribution Analysis

PBMCs were barcoded using the Cell-ID 20-plex PD Barcoding Kit (Fluidigm), enabling simultaneous staining and acquisition of more than 15 samples per run. To minimize inter-run variability, identical control samples from one RA patient and one healthy control were included in every CyTOF acquisition. Inter-batch normalization was then performed using the CytoNorm algorithm, implemented as a FlowJo plugin.

To assess whether T cell clustering patterns were biased by sample identity or acquisition batch, we performed permutation-based multivariate analysis of variance (PERMANOVA) using the adonis2 function in the vegan R package. Two models were tested: one with randomly grouped samples (n = 5 groups, balanced by immunological status), and another grouping samples by immunological status (SP-RA, SN-RA, HC). Euclidean distance matrices were calculated from the normalized CyTOF expression data.

In addition, Principal Component Analysis (PCA) was performed using the same dataset used for clustering, with the prcomp function in R. PCA plots were generated to visualize variance attributable to technical batch (colored by acquisition batch) and to biological status (colored by immunological group). No clustering was observed by batch, whereas group-wise differences were apparent, with healthy controls forming a tighter cluster compared to RA samples, which showed greater immunophenotypic heterogeneity (Supplementary Figure 2).

2.3.5 Software and Tools

- FlowJo v10.8.1: For gating and preprocessing.
- CytoNorm v1.2.3: For data normalization.
- FlowSOM v3.0.18: For clustering.

2.4 Feature Selection and Discriminative T Cell Cluster Identification Using Adaptive Least Absolute Shrinkage and Selection Operator

This study investigated the phenotypic differences between ACCP-Ab+ and ACCP-Ab− peripheral blood T cell clusters in patients with RA, utilizing rigorous computational methodologies within the R environment. The RA dataset was segmented to facilitate a comparative analysis between SP-RA (ACCP-Ab+ and RF+, n = 16) and SN-RA (ACCP-Ab− and RF−) groups.

A uniform analytical approach was applied across three datasets—gating-TCS-DS, FSM-TCL-DS, and FSM-ATCL-DS—comprising immunophenotypic data from 33 peripheral blood T cell samples in the two RA patient groups, differentiated by the presence ACCP-Ab. Prior to analysis, each dataset underwent normalization to ensure uniform scale and variance. Specifically, T cell cluster data were normalized using the centered log-ratio transformation, a standard method for compositional data analysis.

2.4.1 Leave-one-out Cross-validation Analysis

Identification of the most informative dataset and variables capable of distinguishing between SP-RA and SN-RA was a critical step. The analysis integrated several procedures into each cycle of leave-one-out cross-validation (LOOCV) conducted 33 times:

1) Background Factor Correction: Propensity score was calculated to address potential confounding variables, including patient sex, age, symptom duration, NSAID intake, and DAS28-CRP. Subsequently, inverse probability weighting (IPW) was applied to balance covariate distribution between groups, ensuring a robust foundation for analysis.

2) Adaptive least absolute shrinkage and selection operator (adaptive LASSO): The glmnet package was used for initial ridge regression analysis with cv.glmnet in a 10-fold cross-validation setting for binomial distribution, setting alpha to zero. This initial stage determined penalty factors that were subsequently used in adaptive LASSO, adjusted for binomial distribution with an alpha of one. We selected the optimal lambda that minimized the mean cross-validated error, preferring the lambda one standard error from the minimum (lambda.1se) to ensure a balance between model simplicity, predictive accuracy, and overfitting prevention.

3) Predictive Modeling: Variables identified through adaptive LASSO were used to compute the predicted probability of ACCP-Ab positivity for the excluded patient in each LOOCV cycle. We compared predictions to actual clinical classifications of SN-RA and SP-RA, applying a decision threshold of 0.5 to classify outcomes. This methodology allowed for direct model accuracy assessments per LOOCV cycle, contributing to a confusion matrix for detailed evaluation of precision and predictive performance. LOOCV was chosen for its robustness in handling small datasets by maximizing data utilization, because it rigorously tests model performance against individual patient sample, thereby minimizing estimation bias.

2.4.2 Post-LOOCV Analysis

After completing all LOOCV cycles, we assessed datasets to determine the one with the highest overall predictive accuracy by analyzing confusion matrices generated at the optimal lambda for each dataset. These matrices helped identify the most reliable predictions. T cell clusters consistently marked as significant in >50% of the LOOCV iterations within the selected dataset were designated as discriminative T cell clusters (D-TCLs).

The impact of IPW on bias reduction was visually inspected through violin plots of propensity score distributions, supplemented by a weighted Mann–Whitney *U* test to rigorously evaluate differences between the groups. Significant T cell clusters within the selected adaptive LASSO model were highlighted by estimating coefficients, focusing on those with non-zero coefficients as robust discriminative indicators between SP-RA and SN-RA. All analyses and visualizations were conducted using R version 4.3.1 and R packages, including glmnet, caret, boot, cowplot, and ggplot2, to ensure robust computational rigor and a clear graphical representation of our findings.

2.5 Weighted Comparative Analysis of T Cell Cluster Distributions in Patients with SP-RA and SN-RA

To analyze differences in T cell cluster distributions between SP-RA and SN-RA groups, we conducted weighted Mann–Whitney *U* tests. This analysis utilized a dataset comprising RA patient samples, focusing on particular T cell clusters, in addition to relevant patient background variables, including age, sex, symptom duration, DAS28-CRP, and NSAID intake.

Propensity score modeling and IPW weight calculations were performed using the twang package (version 2.6), with the coin (version 1.4-3) and dplyr (version 1.1.2) packages supporting the analysis. The propensity score model, based on these background variables, facilitated the creation of a weighted dataset by replicating rows according to the IPW weights. A traditional Mann–Whitney *U* test was subsequently applied to each TCL variable within this weighted dataset, enabling a comprehensive and unbiased comparison between the SP-RA and SN-RA groups, with adjustments for IPW. Additionally, weighted median values for each TCL were calculated to for a nuanced understanding of the data. This was achieved by extending the dataset according to IPW weights and then computing the median of each cluster for the expanded dataset, ensuring that the median values accurately reflected the weighted characteristics of patient samples.

Weighted TCL distributions were visualized using the ggplot2 package (version 3.4.4) in R. Each variable was represented in a scatter plot, with point sizes proportionate to their IPW weight, generating a beeswarm-like plot for intuitive data representation.

2.6 Support Vector Machine-based Validation of D-TCLs and Clinical Benchmarks in Patients with RA

To validate the discriminative power of the identified D-TCLs between SP-RA and SN-RA groups, we employed a support vector machine (SVM) approach. This analysis was enhanced by a bootstrap method with 1000 iterations to ensure comprehensive validation and reproducibility. Each iteration involved resampling with replacement to create bootstrap samples on which the SVM model was trained and hyperparameters fine-tuned. We optimized the SVM hyperparameters—specifically, the

cost and gamma parameters—through a systematic grid search within each iteration utilizing the caret package in R.

The performance of the SVM model was evaluated on out-of-sample data across multiple metrics, including accuracy, predictive positive value, sensitivity, specificity, negative predictive value, F1 score, and area under the receiver operating characteristic curve (AUC-ROC). We conducted statistical analyses of the bootstrap results to obtain not only mean accuracy and confidence intervals but also median and interquartile measures for each performance metric. Visualization of these results was accomplished through violin plots created using the ggplot2 package, which provided a detailed and clear graphical depiction of model performance across various configurations.

Additional Validation Analyses

Permutation Test for SVM Predictive Performance

All statistical analyses were performed in R (version 4.3.1). The permutation test for support vector machine (SVM) performance was implemented using the following R packages and versions:

- SVM modeling: svm() from **e1071** (version 1.7-13)
- Bootstrap sampling and partitioning: createDataPartition() from **caret** (version 6.0-94)
- ROC/AUC calculation: roc() from **pROC** (version 1.18.4)
- Visualization: ggplot() from **ggplot2** (version 3.4.4)

The SVM classifier employed a radial basis function (RBF) kernel. The following procedure was used:

- First, the optimal SVM hyperparameters (cost and gamma) were determined by grid search using the original labels in the complete dataset.
- The observed mean area under the ROC curve (AUC) was calculated by averaging AUCs over 1000 bootstrap samples, each consisting of a stratified random 70/30 split into training and test sets. The SVM was trained on the training set and AUC was computed on the test set.
- For the permutation step, the ACPA status labels were randomly shuffled 1000 times. For each permutation, the mean AUC was recalculated using the same fixed SVM hyperparameters.
- The distribution of permuted mean AUCs was compared to the observed mean AUC, and the permutation p-value was defined as the proportion of permutations for which the mean AUC was greater than or equal to the observed value.

Alternative Classifier Validation

To further assess the robustness and algorithmic independence of the predictive signature, we performed bootstrap validation (n = 1000) using three additional machine learning algorithms—Elastic Net, Random Forest, and XGBoost—in addition to the SVM.

For each bootstrap iteration:

- The dataset was randomly split (70% training, 30% test) using stratified sampling (createDataPartition() from **caret**, version 6.0-94).

- **Elastic Net:**
  Implemented using the **glmnet** package (version 4.1-7). The optimal regularization parameter (lambda) was determined by 3-fold internal cross-validation within the training set using cv.glmnet() (alpha = 0.5, family = "binomial", type.measure = "deviance"). Predictions on the test set were obtained using the optimal lambda, and AUC was calculated as above.

- **Random Forest:**
  Implemented using the **randomForest** package (version 4.7-1.2). The number of variables randomly sampled at each split (mtry) was tuned by 3-fold cross-validation within the training set using train() from **caret** (method = "rf", metric = "ROC"). The final model was trained using the optimal mtry and ntree = 100, and predicted class probabilities on the test set were used for AUC calculation.

- **XGBoost:**
  Implemented using the **xgboost** package (version 1.7.6.1). Fixed parameters were used based on preliminary tuning (max_depth = 3, eta = 0.1, nrounds = 50). The model was trained on the training set using xgb.train(), and predicted probabilities were used to compute the AUC on the test set.

In all cases, the AUC was calculated using the roc() function from the **pROC** package (version 1.18.4). The mean, 2.5th percentile, and 97.5th percentile (95% confidence interval) of the AUC distributions were reported for each classifier.

2.8 Flow cytometric analysis of additional T cell subset characteristics

Peripheral blood mononuclear cells (PBMCs) from RA patients (n = 4–8 per analysis) were isolated using density gradient centrifugation with Ficoll-Paque Plus (GE Healthcare, Uppsala, Sweden) and resuspended in flow cytometry buffer consisting of Hank's Balanced Salt Solution supplemented with 2% heat-inactivated fetal calf serum, 0.05% sodium azide, and 0.5% EDTA. For surface staining, cells were treated with a comprehensive panel of fluorochrome-conjugated antibodies: Brilliant Violet 510 anti-human CD4 (Clone RPA-T4, BioLegend), AmCyan anti-human CD4 (Clone SK3, BD Biosciences, Franklin Lakes, NJ, USA), Alexa Fluor 647 anti-human CCR4 (Clone TG6/CCR4, eBioscience), PerCP Cy5.5 anti-human CD3 (Clone UCHT1, BioLegend), APC-Cy7 anti-human CD25 (Clone M-A251, BD Biosciences), Pacific Blue anti-human CD183 (CXCR3) (Clone G025H7, BioLegend), FITC anti-human CD8 (Clone SK1, BioLegend), and PE anti-human TCRγδ (Clone B1, BioLegend) for 30 minutes at 4°C in dark conditions. For intracellular staining, cells were fixed and permeabilized using the Foxp3 Staining Buffer Set (eBioscience) for Foxp3 and the Transcription Factor Buffer Set (BD Pharmingen) for T-bet, GATA3, and RORγt, with respective PE-conjugated antibodies: T-bet (Clone QA18A24, BioLegend), GATA3 (Clone 16E10A23, BioLegend), RORγt (Clone Q21-559, BD), and Foxp3 (Clone PCH101, eBioscience). After washing, cells were analyzed

on a FACSCanto II flow cytometer (BD Biosciences) using FACSDiva software, and data were further processed using FlowJo software (Tree Star). This comprehensive staining protocol was designed to accurately identify T cell subsets by both chemokine receptor expression and transcription factor profiling, addressing potential variability in immunophenotyping based solely on surface markers.
